# Genetic and genomic insights into dichogamy in Zingiberaceae

**DOI:** 10.1016/j.xplc.2025.101352

**Published:** 2025-05-08

**Authors:** Shanshan Chen, Xiaochang Peng, Ziyan Xie, Mofan Zhang, Aodan Huang, Weibin Wang, Guisheng Xiang, Kaiquan Zhang, Ranran Gao, Baozhong Duan, Wei Sun, Yuanhong Fan, Shilin Chen, Zhichao Xu

**Affiliations:** 1 College of Life Science, Northeast Forestry University, Harbin 150040, China; 2 State Key Laboratory for Quality Ensurance and Sustainable Use of Dao-di Herbs, Institute of Chinese Materia Medica, China Academy of Chinese Medical Sciences, Beijing 100700, China; 3 Key Laboratory of Beijing for Identification and Safety Evaluation of Chinese Medicine, Institute of Chinese Materia Medica, China Academy of Chinese Medical Sciences, Beijing 100700, China; 4 Ministry of Education Key Laboratory for Transboundary Ecosecurity of Southwest China, Yunnan Key Laboratory of Plant Reproductive Adaptation and Evolutionary Ecology, Institute of Biodiversity, School of Ecology and Environmental Science, Yunnan University, Kunming, Yunnan 650504, China; 5 Yunnan Provincial Key Laboratory of Biological Big Data, Yunnan Agricultural University, Kunming, Yunnan 650201, China; 6 College of Agronomy and Biotechnology, Yunnan Agricultural University, Kunming 650500, China; 7 College of Pharmaceutical Science, Dali University, Dali 671000, China; 8 Yunnan Plateau Characteristic Agricultural Industry Research Institute, Kunming 650500, China; 9 Yunnan Aromatic Bioengineering Technology Research Center, Yunnan Agricultural University, Kunming 650500, China; 10 Institute of Herbgenomics, Chengdu University of Traditional Chinese Medicine, Chengdu 611137, China

Dear Editor,

Dichogamy is a temporal reproductive strategy in which male and female reproductive organs mature at different times, preventing self-fertilization and promoting outcrossing to maintain genetic diversity and support evolutionary adaptation (Goodwillie et al., 2005; Lee et al., 2018). Dichogamous species have evolved diverse and complex mating strategies, one of which involves the temporal separation of male and female reproductive phases within a single flower. Dichogamy has two main subtypes: protandry (PA) and protogyny (PG). In the context of a bisexual flower, PA occurs when the stamen matures and releases pollen before the stigma becomes receptive. PG is the reverse process, in which the pistil becomes receptive before the anther releases pollen. These phenomena, which were historically referred to as male-female and female-male sequences, have now been renamed PA and PG, respectively (Li et al., 2002; Li et al., 2001a; Li et al., 2001b). A number of Zingiberaceae species exhibit PA and PG morphs in bisexual flowers through stylar behavior (flexistyly) during flowering to encourage outcrossing; this has been reported extensively (Li et al., 2001a; Li et al., 2001b; Li et al., 2002; Endress, 2020). In brief, the PA morph releases pollen in the morning, when the stigma of the PG morph descends to facilitate pollen reception. After noon, the anthers of the PG morph release pollen, its stigma moves away from the receptive position, and the stigma of the PA morph descends to facilitate capture of pollen from the PG morph (Figure 1A). Flexistyly shows notable genus specificity within the Zingiberaceae; for instance, *Alpinia* (Li et al., 2001a), *Lanxangia* (Xiaolong et al., 1995; Zhang et al., 2003; Takano et al., 2005; Ying-Qiang et al., 2005), and *Amomum* (Ren et al., 2007) exhibit both PA and PG morphs, whereas *Wurfbainia* and *Etlingera* display only PA morphs (Kress et al., 2005). However, despite extensive studies, the genetic and molecular mechanisms that regulate dichogamy remain unclear. Here, *Lanxangia tsaoko*, which exhibits both PA and PG morphs at the population level (Figure 1A), and *Wurfbainia villosa*, which has only PA morphs, were used to investigate the genetic mechanisms underlying dichogamy within Zingiberaceae species.

**Figure 1:**
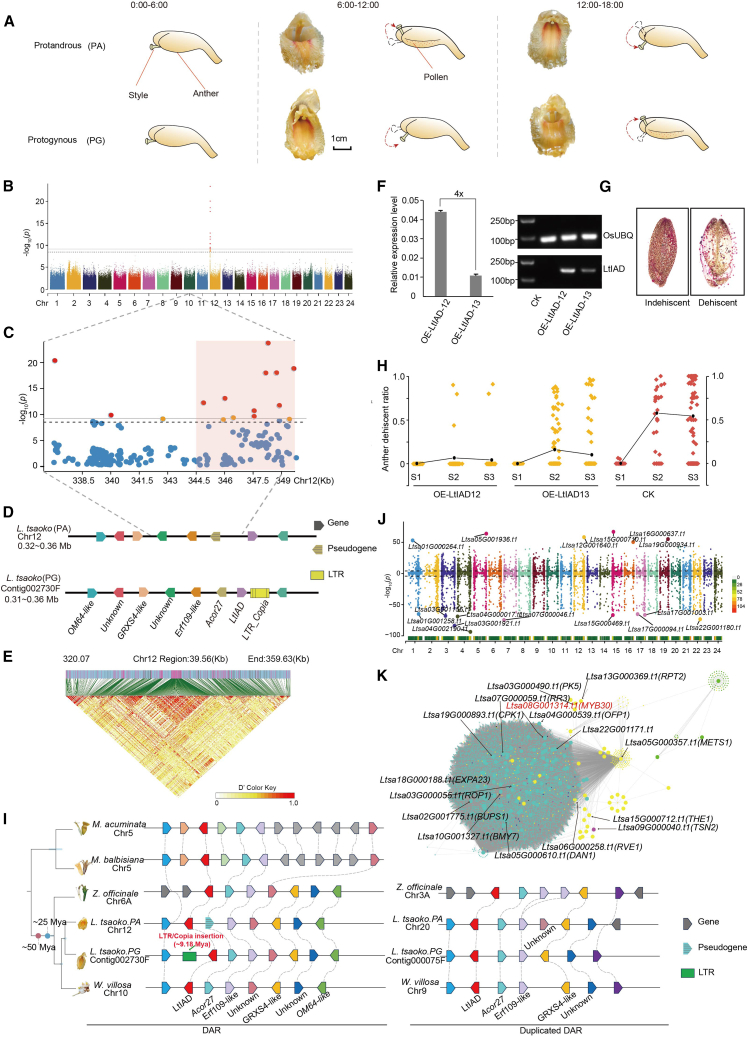
Identification and evolution of DARs in Zingiberaceae and functional validation of *LtIAD*. **(A)** Stylar movement and anther dehiscence in the PA (protandry) and PG (protogyny) morphs of *L. tsaoko*. **(B)** Manhattan plot of genome-wide SNP (single-nucleotide polymorphism) markers associated with the PA morph. The strongest associated SNP was identified on chromosome 12. The dashed horizontal black line and the dotted line represent the significant *p* value threshold of 8.44, calculated as 0.05/n. **(C)** Manhattan plot of the target region on chromosome 12. Red squares indicate regions with significant associations. **(D)** Localization of genes identified through GWAS (genome-wide association study) analysis. The differently colored squares in the diagram represent distinct genes. **(E)** LD block plot. The red triangles represent regions with high linkage disequilibrium (LD). **(F)** Overexpression of *LtIAD* in transgenic lines measured by RT–qPCR and RT–PCR, with data presented as mean ± SD of three technical replicates. **(G)** The dehiscence condition of the anther wall and the red color of the pollen after staining in *O. sativa*. **(H)** Dehiscence condition of the anther wall in the OE-*LtIAD-12* and OE-*LtIAD-13* overexpression lines. More than 20 biological replicates were measured at each stage, and each point represents an individual replicate. The *y* axis indicates the anther dehiscence ratio. Black dots indicate the anther number which dehiscent area over 50% in each line. **(I)** Partial phylogenetic tree of Musaceae and Zingiberaceae species (left) and their corresponding duplicated genomic regions (DARs) (right). Light blue bars at the nodes represent 95% confidence intervals for the estimated divergence times. Whole-genome duplication events in Zingiberaceae are marked by red and blue dots. In each DAR, blocks of the same color represent syntenic gene pairs. **(J)** Transcriptome-wide association study analysis. The green heatmap at the bottom represents gene density, with the dashed line marking the significance threshold of *p* ≤ 0.01. The dots at the top indicate different genes on each chromosome, and the red dots highlight genes jointly identified by the transcriptome-wide association study and the eQTL analysis. **(K)** Co-expression network of genes from the nine gene sets in Supplemental Figure 19. Labeled nodes represent core genes with high connectivity or key gene functions within the network.

We first assembled high-quality chromosome-level genomes of *L. tsaoko* and *W. villosa*. In a previously published version of the *L. tsaoko* genome, the sequenced individual displayed the PG phenotype. We therefore selected an *L. tsaoko* individual with the PA phenotype for genome sequencing. The haploid genome of *L. tsaoko*, totaling 2.08 Gb and anchored onto 24 pseudo-chromosomes with 94.2% benchmarking universal single-copy orthologs (BUSCO) completeness, was assembled using 314 Gb (∼150×) of Illumina sequencing data, 203.57 Gb (∼97×) of PacBio long reads, and 247 Gb (∼118×) of Hi-C data (Supplemental Figure 1; Supplemental Tables 1–5). Similarly, a high-quality, 2.69-Gb pseudo-chromosome genome (2n = 48) was assembled for *W. villosa*, which also exhibits the PA phenotype (Supplemental Figure 4; Supplemental Tables 6–9). After annotating and masking repetitive elements, we predicted 41 278 protein-coding genes in the *L. tsaoko* genome and 38 256 in the *W. villosa* genome (Supplemental Tables 10–15). Phylogenetic analysis of orthologous genes indicated that *W. villosa*, *L. tsaoko*, and *Zingiber officinale* formed a cluster within the Zingiberaceae branch, and the divergence time between *L. tsaoko* and *W. villosa* was estimated to be approximately 7.96 million years ago (mya) (Supplemental Figures 5–7). Genomic collinearity and the distributions of synonymous substitutions per synonymous site for paralogous and orthologous genes indicated that all tested Zingiberaceae species shared two rounds of whole-genome duplication (∼25 and ∼50 mya) (Supplemental Figures 8–12).

To identify genetic loci related to dichogamy in *L. tsaoko*, we re-sequenced the genomes of 197 diploid *L. tsaoko* accessions, including 98 individuals with PG morphs and 99 individuals with PA morphs, achieving an average sequencing coverage of 21.1× (Supplemental Table 1). A genome-wide association study (GWAS) using the PA-morph reference genome revealed a prominent SNP signal block on chromosome 12, with the top-ranked SNP (12_348188, *p* = 3.768273E−24), spanning a genomic region of approximately 13 kb (336 954–349 548 bp; Figure 1B and 1C). Similarly, using a previously published genome (PG morph) as a reference, the GWAS analysis also identified a region of high linkage disequilibrium (LD) near the beginning of contig 002730F (*p* = 3.196953E−26) with a length of 9 kb (329 491–339 921) (Supplemental Figures 13 and 14). The high-LD regions from the PA and PG genomes showed high homology in a collinearity analysis and were designated the dichogamy-associated region (DAR) (Figure 1D and 1E). Six pairs of syntenic genes were annotated in the DARs of the PA and PG morphs, including *ERF109*-like, *GRXS4*-like, and *OM64*-like genes and three unknown genes. However, despite the overall high similarity between these regions, significant structural variation was identified, specifically near *Ltsa12G000047.t1*. Further analysis revealed that the expression of *Ltsa12G000047.t1* was significantly higher in flowers of PG morphs than PA morphs (Supplemental Figure 15). RT–qPCR showed that *Ltsa12G000047.t1* was most highly expressed in anthers (in both PA and PG). Therefore, we speculate that this gene may affect pollen dehiscence (Supplemental Figure 16). In addition, a complete long terminal repeat (LTR)-Copia insertion was observed downstream of *Ltsa12G000047.t1* in the PG-morph genome, leading us to speculate that this insertion might be responsible for the differences in gene expression.

We next overexpressed *Ltsa12G000047.t1* in *Oryza sativa* to investigate its role in flowering. Two overexpression lines, *OE-Ltsa12G000047.t1-12* and *OE-Ltsa12G000047.t1-13*, were generated and confirmed using semi-quantitative PCR and real-time PCR (Figure 1F and Supplemental Figure 17). On the basis of the flowering characteristics of rice, we divided the flowering process into three stages, S1–S3 (Supplemental Figure 18), and examined anther dehiscence at each stage (Figure 1G). Anthers with more than 50% dehiscence were counted for each line. The results showed that the anther dehiscence ratio was significantly lower in *OE-Ltsa12G000047.t1-12* and *OE-Ltsa12G000047.t1-13* than in the wild-type line (Figure 1H). These findings indicate that *Ltsa12G000047.t1* directly or indirectly inhibits anther dehiscence during flowering stages, thereby affecting pollination. Given its potential function, we designated this gene *LtIAD* (influence anther dehiscence) and renamed the overexpression lines *OE-LtIAD-12* and *OE-LtIAD-13*.

To explore the evolutionary origins of the DAR, we performed comparative genomic analyses of the *Musa acuminata*, *Musa balbisiana*, *Z. officinale* (PA), *L. tsaoko* (PA and PG), and *W. villosa* (PA) genomes. The syntenic DAR loci were found to be conserved among the Zingiberaceae and *Musa genomes*, together with abundant LTR insertions and gene rearrangements. The six DAR-localized genes are conserved in Zingiberaceae species, but there are five genes inserted between the ERF109-like gene and the upstream unknown gene in *Musa*, and three genes (*GRXS4-like*, *OM64-like*, and an unknown gene) have been lost upstream of the unknown gene (Figure 1I and Supplemental Figure 19). Furthermore, all tested Zingiberaceae species contained remnants of two paralogous fragments of the DAR, suggesting that the shared WGD event around 50 mya duplicated the DAR; the function of the duplicated DAR is unknown. By contrast, the PG morph of *L. tsaoko* exhibited a single LTR/Copia insertion downstream of *LtIAD*, estimated to have occurred approximately ∼9.18 mya (Figure 1I). We hypothesize that this LTR insertion may play a crucial role in dichogamy-related phenotypic differentiation.

To identify genes associated with key SNP loci from the GWAS, we performed an additional analysis (Figure 1J; Supplemental Tables 19 and 20), which led to the identification of 176 candidate genes. With these candidate genes as the core, we constructed a weighted correlation network analysis based on the expression profiles of all genes, and 138 target genes were identified (Supplemental Table 21). The turquoise module in the weighted correlation network analysis exhibited the most significant gene signifificance-module membership correlation, followed by the yellow and magenta modules (Supplemental Figure 20). Subsequently, we identified the homologs of each target gene in *Arabidopsis thaliana* and found that the co-expression network was enriched in genes associated with phytohormones, including auxin (*Ltsa06G000258.t1*, *Resolvin E1*, *RVE1*), as well as genes related to cell elongation (*Ltsa04G000539.t1*, OVATE family protein, OFP1), light stress response, and pollen germination (*Ltsa08G001314.t1*, *MYB DOMAIN PROTEIN*, *MYB30*) (Figure 1K). We also identified genes related to cell wall integrity, such as *Ltsa15G000712.t1* (*THESEUS1*, *THE1*) and *Ltsa02G001775.t1* (*Buddha’s Paper Seal 1*, *BUPS1*). These genes and their associated pathways likely play crucial roles in the dichogamy of *L. tsaoko*.

In conclusion, our study provides high-quality chromosome-level genomes for *L. tsaoko* and *W. villosa* together with resequencing data for 197 *L. tsaoko* samples and accompanying transcriptome data, which provide essential information for studies of these two economically important crops. Moreover, our findings reveal an LD region responsible for dichogamous flower morphs, and a novel gene, *IAD*, that may be related to anther dehiscence. Our study also provides insights into the origin and evolutionary dynamics of the molecular mechanisms that govern dichogamy regulation in angiosperms.

## Data and code availability

The raw sequencing data are available in the Sequence Read Archive, Biological Research Project Data, NCBI repository (accessions PRJNA608086 and PRJNA608081). The assembled genome and gene structures of *L. tsaoko and W. villosa* have been deposited in Figshare (https://doi.org/10.6084/m9.figshare.27600624.v1).

## Funding

This work was supported by the Scientific and Technological Innovation Project of the 10.13039/501100005892China Academy of Chinese Medical Sciences (CI2023E002), the YEFICRC project of Yunnan provincial key programs (2019ZG009), and the National Science and Technology Resource Sharing Service Platform Project (NTPGRC2023-01).

## Acknowledgments

No conflict of interest is declared.

## Author contributions

Shi Chen, Z. Xu, and Y.F. designed the experiments. Z. Xie, A.H., M.Z., X.P., K.Z., W.S., R.G., and B.D. performed the experiments. Shan Chen, W.W., and G.X. analyzed the data. Shan Chen and X.P. wrote the manuscript. Shan Chen and Z X. reviewed，and edited the manuscript. All authors contributed to the article and approved the final manuscript.

## Supplemental information

Supplemental Data 1. Supplemental Tables 1–22

Document S2. Article plus supplemental information
