# Supplementary material for: Genetic and genomic insights into dichogamy in Zingiberaceae
Source: Plant Commun. 2025 May 8;6(7):101352. doi: 10.1016/j.xplc.2025.101352 (PMC12281188; doi:10.1016/j.xplc.2025.101352)
Supplement: Document S2. Article plus supplemental information [file mmc3.pdf]

Plant Communications
Correspondence

CellPress
Partner Journal

Genetic and genomic insights into dichogamy in Zingiberaceae

Dear Editor,

Dichogamy is a temporal reproductive strategy in which male and female reproductive organs mature at different times, preventing self-fertilization and promoting outcrossing to maintain genetic diversity and support evolutionary adaptation (Goodwillie et al., 2005; Lee et al., 2018). Dichogamous species have evolved diverse and complex mating strategies, one of which involves the temporal separation of male and female reproductive phases within a single flower. Dichogamy has two main subtypes: protandry (PA) and protogyny (PG). In the context of a bisexual flower, PA occurs when the stamen matures and releases pollen before the stigma becomes receptive. PG is the reverse process, in which the pistil becomes receptive before the anther releases pollen. These phenomena, which were historically referred to as male-female and female-male sequences, have now been renamed PA and PG, respectively (Li et al., 2002; Li et al., 2001a; Li et al., 2001b). A number of Zingiberaceae species exhibit PA and PG morphs in bisexual flowers through stylar behavior (flexistyly) during flowering to encourage outcrossing; this has been reported extensively (Li et al., 2001a; Li et al., 2001b; Li et al., 2002; Endress, 2020). In brief, the PA morph releases pollen in the morning, when the stigma of the PG morph descends to facilitate pollen reception. After noon, the anthers of the PG morph release pollen, its stigma moves away from the receptive position, and the stigma of the PA morph descends to facilitate capture of pollen from the PG morph (Figure 1A). Flexistyly shows notable genus specificity within the Zingiberaceae; for instance, *Alpinia* (Li et al., 2001a), *Lanxangia* (Xiaolong et al., 1995; Zhang et al., 2003; Takano et al., 2005; Ying-Qiang et al., 2005), and *Amomum* (Ren et al., 2007) exhibit both PA and PG morphs, whereas *Wurfbainia* and *Etlingera* display only PA morphs (Kress et al., 2005). However, despite extensive studies, the genetic and molecular mechanisms that regulate dichogamy remain unclear. Here, *Lanxangia tsaoko*, which exhibits both PA and PG morphs at the population level (Figure 1A), and *Wurfbainia villosa*, which has only PA morphs, were used to investigate the genetic mechanisms underlying dichogamy within Zingiberaceae species.

We first assembled high-quality chromosome-level genomes of *L. tsaoko* and *W. villosa*. In a previously published version of the *L. tsaoko* genome, the sequenced individual displayed the PG phenotype. We therefore selected an *L. tsaoko* individual with the PA phenotype for genome sequencing. The haploid genome of *L. tsaoko*, totaling 2.08 Gb and anchored onto 24 pseudo-chromosomes with 94.2% benchmarking universal single-copy orthologs (BUSCO) completeness, was assembled using 314 Gb (~150×) of Illumina sequencing data, 203.57 Gb (~97×) of PacBio long reads, and 247 Gb (~118×) of Hi-C data (Supplemental Figure 1; Supplemental Tables 1–5). Similarly, a high-quality, 2.69-Gb pseudo-chromosome genome (2n = 48) was assembled for *W. villosa*, which also exhibits the PA phenotype (Supplemental Figure 4; Supplemental Tables 6–9). After annotating and masking repetitive elements, we predicted 41 278 protein-coding genes in the *L. tsaoko* genome and 38 256 in the *W. villosa* genome (Supplemental Tables 10–15). Phylogenetic analysis of orthologous genes indicated that *W. villosa*, *L. tsaoko*, and *Zingiber officinale* formed a cluster within the Zingiberaceae branch, and the divergence time between *L. tsaoko* and *W. villosa* was estimated to be approximately 7.96 million years ago (mya) (Supplemental Figures 5–7). Genomic collinearity and the distributions of synonymous substitutions per synonymous site for paralogous and orthologous genes indicated that all tested Zingiberaceae species shared two rounds of whole-genome duplication (~25 and ~50 mya) (Supplemental Figures 8–12).

To identify genetic loci related to dichogamy in *L. tsaoko*, we resequenced the genomes of 197 diploid *L. tsaoko* accessions, including 98 individuals with PG morphs and 99 individuals with PA morphs, achieving an average sequencing coverage of 21.1× (Supplemental Table 1). A genome-wide association study (GWAS) using the PA-morph reference genome revealed a prominent SNP signal block on chromosome 12, with the top-ranked SNP (12_348188, p = 3.768273E−24), spanning a genomic region of approximately 13 kb (336 954–349 548 bp; Figure 1B and 1C). Similarly, using a previously published genome (PG morph) as a reference, the GWAS analysis also identified a region of high linkage disequilibrium (LD) near the beginning of contig 002730F (p = 3.196953E−26) with a length of 9 kb (329 491–339 921) (Supplemental Figures 13 and 14). The high-LD regions from the PA and PG genomes showed high homology in a collinearity analysis and were designated the dichogamy-associated region (DAR) (Figure 1D and 1E). Six pairs of syntenic genes were annotated in the DARs of the PA and PG morphs, including *ERF109*-like, *GRXS4*-like, and *OM64*-like genes and three unknown genes. However, despite the overall high similarity between these regions, significant structural variation was identified, specifically near *Ltsa12G000047.t1*. Further analysis revealed that the expression of *Ltsa12G000047.t1* was significantly higher in flowers of PG morphs than PA morphs (Supplemental Figure 15). RT–qPCR showed that *Ltsa12G000047.t1* was most highly expressed in anthers (in both PA and PG). Therefore, we speculate that this gene may affect pollen dehiscence (Supplemental Figure 16). In addition, a complete long terminal repeat (LTR)-Copia insertion was observed downstream of *Ltsa12G000047.t1* in the PG-morph genome, leading us to speculate that this insertion might be responsible for the differences in gene expression.

We next overexpressed *Ltsa12G000047.t1* in *Oryza sativa* to investigate its role in flowering. Two overexpression lines,

© 2025 The Author(s). Published by Elsevier Inc. on behalf of CAS Center for Excellence in Molecular Plant Sciences, Chinese Academy of Sciences, and Chinese Society for Plant Biology.
This is an open access article under the CC BY license (http://creativecommons.org/licenses/by/4.0/).

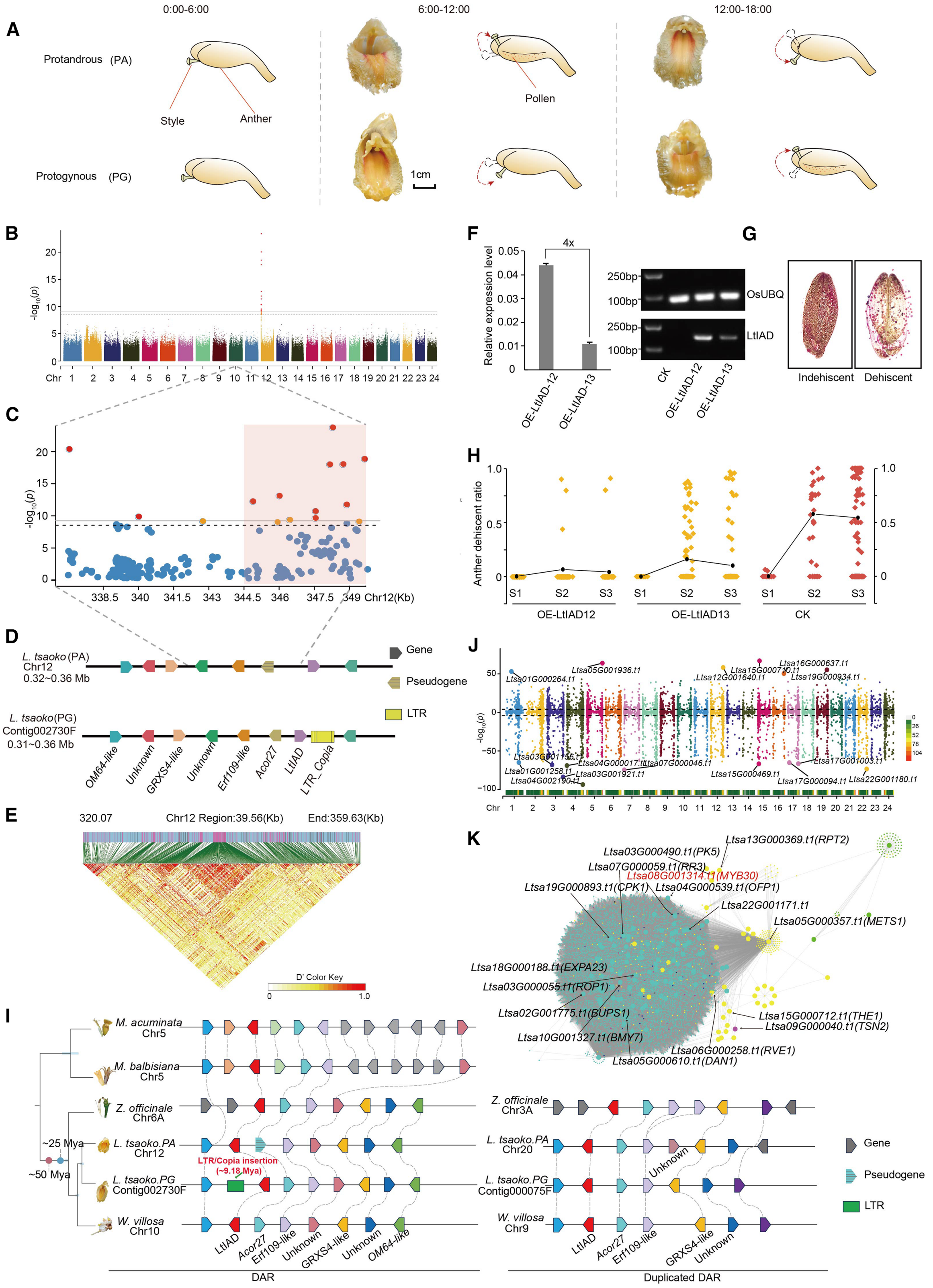

Figure 1. Identification and evolution of DARs in Zingiberaceae and functional validation of *LtIAD*.

(A) Stylar movement and anther dehiscence in the PA (protandry) and PG (protogyny) morphs of *L. tsaoko*.

(B) Manhattan plot of genome-wide SNP (single-nucleotide polymorphism) markers associated with the PA morph. The strongest associated SNP was identified on chromosome 12. The dashed horizontal black line and the dotted line represent the significant *p* value threshold of 8.44, calculated as 0.05/n.

(legend continued on next page)

OE-Ltsa12G000047.t1-12 and *OE-Ltsa12G000047.t1-13*, were generated and confirmed using semi-quantitative PCR and real-time PCR (Figure 1F and Supplemental Figure 17). On the basis of the flowering characteristics of rice, we divided the flowering process into three stages, S1–S3 (Supplemental Figure 18), and examined anther dehiscence at each stage (Figure 1G). Anthers with more than 50% dehiscence were counted for each line. The results showed that the anther dehiscence ratio was significantly lower in *OE-Ltsa12G000047.t1-12* and *OE-Ltsa12G000047.t1-13* than in the wild-type line (Figure 1H). These findings indicate that *Ltsa12G000047.t1* directly or indirectly inhibits anther dehiscence during flowering stages, thereby affecting pollination. Given its potential function, we designated this gene *LtIAD* (influence anther dehiscence) and renamed the overexpression lines *OE-LtIAD-12* and *OE-LtIAD-13*.

To explore the evolutionary origins of the DAR, we performed comparative genomic analyses of the *Musa acuminata*, *Musa balbisiana*, *Z. officinale* (PA), *L. tsaoko* (PA and PG), and *W. villosa* (PA) genomes. The syntenic DAR loci were found to be conserved among the Zingiberaceae and *Musa genomes*, together with abundant LTR insertions and gene rearrangements. The six DAR-localized genes are conserved in Zingiberaceae species, but there are five genes inserted between the ERF109-like gene and the upstream unknown gene in *Musa*, and three genes (*GRXS4-like*, *OM64-like*, and an unknown gene) have been lost upstream of the unknown gene (Figure 1I and Supplemental Figure 19). Furthermore, all tested Zingiberaceae species contained remnants of two paralogous fragments of the DAR, suggesting that the shared WGD event around 50 mya duplicated the DAR; the function of the duplicated DAR is unknown. By contrast, the PG morph of *L. tsaoko* exhibited a single LTR/Copia insertion downstream of *LtIAD*, estimated to have occurred approximately ∼9.18 mya (Figure 1I). We hypothesize that this LTR insertion may play a crucial role in dichogamy-related phenotypic differentiation.

To identify genes associated with key SNP loci from the GWAS, we performed an additional analysis (Figure 1J; Supplemental Tables 19 and 20), which led to the identification of 176 candidate genes. With these candidate genes as the core, we constructed a weighted correlation network analysis based on the expression profiles of all genes, and 138 target genes were identified (Supplemental Table 21). The turquoise module in the weighted correlation network analysis exhibited the most significant gene signifificance-module membership correlation, followed by the yellow and magenta modules (Supplemental Figure 20). Subsequently, we identified the homologs of each target gene in *Arabidopsis thaliana* and found that the co-expression network was enriched in genes associated with phytohormones, including auxin (*Ltsa06G000258.t1*, *Resolvin E1*, *RVE1*), as well as genes related to cell elongation (*Ltsa04G000539.t1*, OVATE family protein, OFP1), light stress response, and pollen germination (*Ltsa08G001314.t1*, *MYB DOMAIN PROTEIN*, *MYB30*) (Figure 1K). We also identified genes related to cell wall integrity, such as *Ltsa15G000712.t1* (*THESEUS1*, *THE1*) and *Ltsa02G001775.t1* (*Buddha's Paper Seal 1*, *BUPS1*). These genes and their associated pathways likely play crucial roles in the dichogamy of *L. tsaoko*.

In conclusion, our study provides high-quality chromosome-level genomes for *L. tsaoko* and *W. villosa* together with re-sequencing data for 197 *L. tsaoko* samples and accompanying transcriptome data, which provide essential information for studies of these two economically important crops. Moreover, our findings reveal an LD region responsible for dichogamous flower morphs, and a novel gene, *IAD*, that may be related to anther dehiscence. Our study also provides insights into the origin and evolutionary dynamics of the molecular mechanisms that govern dichogamy regulation in angiosperms.

DATA AND CODE AVAILABILITY

The raw sequencing data are available in the Sequence Read Archive, Biological Research Project Data, NCBI repository (accessions PRJNA608086 and PRJNA608081). The assembled genome and gene structures of *L. tsaoko and W. villosa* have been deposited in Figshare (https://doi.org/10.6084/m9.figshare.27600624.v1).

FUNDING

This work was supported by the Scientific and Technological Innovation Project of the China Academy of Chinese Medical Sciences (CI2023E002), the YEFICRC project of Yunnan provincial key programs (2019ZG009), and the National Science and Technology Resource Sharing Service Platform Project (NTPGRC2023-01).

(C) Manhattan plot of the target region on chromosome 12. Red squares indicate regions with significant associations.
(D) Localization of genes identified through GWAS (genome-wide association study) analysis. The differently colored squares in the diagram represent distinct genes.
(E) LD block plot. The red triangles represent regions with high linkage disequilibrium (LD).
(F) Overexpression of *LtIAD* in transgenic lines measured by RT–qPCR and RT–PCR, with data presented as mean ± SD of three technical replicates.
(G) The dehiscence condition of the anther wall and the red color of the pollen after staining in *O. sativa*.
(H) Dehiscence condition of the anther wall in the OE-*LtIAD-12* and OE-*LtIAD-13* overexpression lines. More than 20 biological replicates were measured at each stage, and each point represents an individual replicate. The *y* axis indicates the anther dehiscence ratio. Black dots indicate the anther number which dehiscent area over 50% in each line.
(I) Partial phylogenetic tree of Musaceae and Zingiberaceae species (left) and their corresponding duplicated genomic regions (DARs) (right). Light blue bars at the nodes represent 95% confidence intervals for the estimated divergence times. Whole-genome duplication events in Zingiberaceae are marked by red and blue dots. In each DAR, blocks of the same color represent syntenic gene pairs.
(J) Transcriptome-wide association study analysis. The green heatmap at the bottom represents gene density, with the dashed line marking the significance threshold of $p \leq 0.01$. The dots at the top indicate different genes on each chromosome, and the red dots highlight genes jointly identified by the transcriptome-wide association study and the eQTL analysis.
(K) Co-expression network of genes from the nine gene sets in Supplemental Figure 19. Labeled nodes represent core genes with high connectivity or key gene functions within the network.

ACKNOWLEDGMENTS
No conflict of interest is declared.

AUTHOR CONTRIBUTIONS
Shi Chen, Z. Xu, and Y.F. designed the experiments. Z. Xie, A.H., M.Z., X. P., K.Z., W.S., R.G., and B.D. performed the experiments. Shan Chen, W. W., and G.X. analyzed the data. Shan Chen and X.P. wrote the manuscript. Shan Chen and Z X. reviewed , and edited the manuscript. All authors contributed to the article and approved the final manuscript.

SUPPLEMENTAL INFORMATION
Supplemental information is available at *Plant Communications Online*.

Received: January 13, 2025
Revised: March 21, 2025
Accepted: May 5, 2025
Published: May 8, 2025

Shanshan Chen[1,2,3,11], Xiaochang Peng[4,11], Ziyan Xie[1,11], Mofan Zhang[1], Aodan Huang[4], Weibin Wang[5], Guisheng Xiang[6], Kaiquan Zhang[1], Ranran Gao[2,3], Baozhong Duan[7], Wei Sun[2,3], Yuanhong Fan[6,8,9,], Shilin Chen[3,10,*] and Zhichao Xu[1,*]*

[1]College of Life Science, Northeast Forestry University, Harbin 150040, China
[2]State Key Laboratory for Quality Ensurance and Sustainable Use of Dao-di Herbs, Institute of Chinese Materia Medica, China Academy of Chinese Medical Sciences, Beijing 100700, China
[3]Key Laboratory of Beijing for Identification and Safety Evaluation of Chinese Medicine, Institute of Chinese Materia Medica, China Academy of Chinese Medical Sciences, Beijing 100700, China
[4]Ministry of Education Key Laboratory for Transboundary Ecosecurity of Southwest China, Yunnan Key Laboratory of Plant Reproductive Adaptation and Evolutionary Ecology, Institute of Biodiversity, School of Ecology and Environmental Science, Yunnan University, Kunming, Yunnan 650504, China
[5]Yunnan Provincial Key Laboratory of Biological Big Data, Yunnan Agricultural University, Kunming, Yunnan 650201, China
[6]College of Agronomy and Biotechnology, Yunnan Agricultural University, Kunming 650500, China
[7]College of Pharmaceutical Science, Dali University, Dali 671000, China
[8]Yunnan Plateau Characteristic Agricultural Industry Research Institute, Kunming 650500, China
[9]Yunnan Aromatic Bioengineering Technology Research Center, Yunnan Agricultural University, Kunming 650500, China
[10]Institute of Herbgenomics, Chengdu University of Traditional Chinese Medicine, Chengdu 611137, China
[11]These authors contributed equally to this article.
***Correspondence: Yuanhong Fan (2017666@ynau.edu.cn), Shilin Chen (slchen@cdutcm.edu.cn), Zhichao Xu (zcxu@nefu.edu.cn)**
https://doi.org/10.1016/j.xplc.2025.101352

REFERENCES
Endress, P.K. (2020). Structural and temporal modes of heterodichogamy and similar patterns across angiosperms. Bot. J. Linn. Soc. **193**:5–18.

Goodwillie, C., Kalisz, S., and Eckert, C.G. (2005). The evolutionary enigma of mixed mating systems in plants: occurrence, theoretical explanations, and empirical evidence. Annu. Rev. Ecol. Evol. Syst. **36**:47–79.

Kress, W.J., Liu, A.Z., Newman, M., and Li, Q.J. (2005). The molecular phylogeny of *Alpinia* (Zingiberaceae): a complex and polyphyletic genus of gingers. Am. J. Bot. **92**:167–178.

Lee, H., Kang, H., and Park, W.-G. (2018). A rare duodichogamous flowering system in monoecious *Toona sinensis* (Meliaceae). J. Ecol. Environ. **42**:7–10.

Li, Q.-J., Kress, W.J., Xu, Z.-F., Xia, Y.-M., Zhang, L., Deng, X.-B., and Gao, J.-Y. (2002). Mating system and stigmatic behaviour during flowering of *Alpinia kwangsiensis* (Zingiberaceae). Plant Systemat. Evol. **232**:123–132.

Li, Q.-J., Xu, Z.-F., Kress, W.J., Xia, Y.-M., Zhang, L., Deng, X.-B., Gao, J.-Y., and Bai, Z.-L. (2001). Flexible style that encourages outcrossing. Nature **410**:432.

Li, Q.-J., Xu, Z.-F., Xia, Y.-M., and Zhang, L. (2001). Study on the flexistyly pollination mechanism in *Alpinia* plants (Zingiberaceae). Acta Bot. Sin. **43**:364–369.

Ren, P.-Y., Liu, M., and Li, Q.-J. (2007). An example of flexistyly in a wild cardamom species (*Amomum maximum* (Zingiberaceae)). Plant Systemat. Evol. **267**:147–154.

Takano, A., Gisil, J., Yusoff, M., and Tachi, T. (2005). Floral and pollinator behaviour of flexistylous Bornean ginger, *Alpinia nieuwenhuizii* (Zingiberaceae). Plant Systemat. Evol. **252**:167–173.

Xiaolong, C., Rongcheng, W., and Ruifu, H. (1995). A preliminary study on the genetic system of *Amomum tsao-ko*. J. Yunnan Univ. **17**:290–297.

Ying-Qiang, W., Dian-Xiang, Z., and Zhong-Yi, C. (2005). Pollination biology of *Alpinia hainanensis* (Zingiberaceae). J. Systemat. Evol. **43**:37.

Zhang, L., Li, Q.-J., Deng, X.-B., Ren, P.-Y., and Gao, J.-Y. (2003). Reproductive biology of *Alpinia blepharocalyx* (Zingiberaceae): another example of flexistyly. Plant Systemat. Evol. **241**:67–76.

Plant Communications, Volume *6*

Supplemental information

Genetic and genomic insights into dichogamy in Zingiberaceae

Shanshan Chen, Xiaochang Peng, Ziyan Xie, Mofan Zhang, Aodan Huang, Weibin Wang, Guisheng Xiang, Kaiquan Zhang, Ranran Gao, Baozhong Duan, Wei Sun, Yuanhong Fan, Shilin Chen, and Zhichao Xu

Supporting information

Article title: Genetic and genomic insights into the dichogamy in Zingiberaceae

Authors: Shanshan Chen[1,2,3,11], Xiaochang Peng[4,11], Ziyan Xie[1,11], Mofan Zhang[1], Aodan Huang[4], Weibin Wang[5], Guisheng Xiang[6], Kaiquan Zhang[1], Ranran Gao[2,3], Baozhong Duan[7], Wei Sun[2,3], Yuanhong Fan[6,8,9,*], Shilin Chen[3,10,*], Zhichao Xu[1,*]

[1]College of Life Science, Northeast Forestry University, Harbin, 150040, China

[2]State Key Laboratory for Quality Ensurance and Sustainable Use of Dao-di Herbs, Institute of Chinese Materia Medica, China Academy of Chinese Medical Sciences, 100700

[3]Key Laboratory of Beijing for Identification and Safety Evaluation of Chinese Medicine, Institute of Chinese Materia Medica, China Academy of Chinese Medical Sciences, 100700 Beijing, China

[4]Ministry of Education Key Laboratory for Transboundary Ecosecurity of Southwest China, Yunnan Key Laboratory of Plant Reproductive Adaptation and Evolutionary Ecology, Institute of Biodiversity, School of Ecology and Environmental Science, Yunnan University, Kunming, 650504, Yunnan, China

[5]Yunnan Provincial Key Laboratory of Biological Big Data, Yunnan Agricultural University, Kunming, Yunnan, 650201, China

[6]College of Agronomy and Biotechnology, Yunnan Agricultural University, Kunming 650500, China

[7]College of Pharmaceutical Science, Dali University, Dali, 671000, China

[8]Yunnan Plateau Characteristic Agricultural Industry Research Institute, Kunming 650500, China

[9]Yunnan Aromatic Bioengineering Technology Research Center, Yunnan Agricultural University, Kunming 650500, China

[10]Institute of Herbgenomics, Chengdu University of Traditional Chinese Medicine, Chengdu 611137, China

[11]These authors contributed equally to this article.

***Corresponding author:** Yuanhong Fan, E-mail: 2017666@ynau.edu.cn, Shilin Chen, E-mail: slchen@cdutcm.edu.cn, Tel: 86-13311151899，Zhichao Xu, E-mail: zcxu@nefu.edu.cn, Tel: 86-18610014204

This file includes:

- **Supplemental Materials and Methods**
- **Supplemental Figures**

Supplemental Figure 1. Heatmap of the Hi-C interaction density between 24 pseudochromosomes of *L. tsaoko*.

Supplemental Figure 2. Syntenic analysis of the published *L. tsaoko* genome and the genome assembled in this article.

Supplemental Figure 3. The survey analysis to estimate the *W. villosum* genome size.

Supplemental Figure 4. Heatmap of the Hi-C interaction density between 24 pseudochromosomes of *W. villosum*.

Supplemental Figure 5. Estimation of divergence times for candidate species using MCMCtree.

Supplemental Figure 6. Expansion and contraction of gene families in 12 angiosperm species using CAFE5.

Supplemental Figure 7. Phylogenetic relationship and WGD events of *L. tsaoko*.

Supplemental Figure 8. Synteny blocks between *L. tsaoko* and *Z. officinale***.**

Supplemental Figure 9. K_S distributions of whole paralogs (grey bars) and anchor-pair paralogs (black bars) for *Z. officinale*.

Supplemental Figure 10. Distribution of synonymous substitution rates (K_S) for the anchored paralogs of *Z. officinale* and the anchored orthologs between *Z. officinale* and *L. tsaoko* and *M. acuminata, M. balbisiana*.

Supplemental Figure 11. K_S distributions of whole paralogs (grey bars) and anchor-pair paralogs (black bars) for *W. villosum*.

Supplemental Figure 12. Substitution-rate-adjusted mixed paralog-ortholog synonymous substitutions per synonymous site (K_S) plot for *W. villosum* and the anchored orthologs between *W. villosum* and *Z. officinale*, *L. tsaoko* and *M. acuminata, M. balbisiana*.

Supplemental Figure 13. Manhattan plot of genome-wide SNP markers associated with dichogam.

Supplemental Figure 14. QQ plot of genome-wide SNP markers associated with dichogam.

Supplemental Figure 15. Heatmap of gene expression in the *L. tsaoko* - *DAR* region of *L. tsaoko* genome.

Supplemental Figure 16. Expression profiling of *LtIAD* in different tissues of *L. tsaoko*.

Supplemental Figure 17. Identification of *LtIAD* overexpression lines by hygromycin (hygromycin amplified fragment size was 557 bp, M: D2000 Marker).

Supplemental Figure 18. The flowering stage are divided into stage 1, stage 2, and stage 3 according to the open condition of palea and lemmal.

Supplemental Figure 19. The evolution of DARs in the six genomes.

Supplemental Figure 20. Co-expression network analysis.

- **Supplemental Materials and Methods**

Plant Materials

The *L. tsaoko* samples were collected from Dashuijing Village (103°35′E, 23°7′N), Pingbian, Yunnan. Three PA morph whole plants were obtained for genome sequencing. In addition, a total of 99 PA morph and 98 PG morph leaf samples were collected for resequencing, and whole flower samples from the same individuals were harvested around 11:00 AM for transcriptome analysis.

The *W. villosum* samples were collected in the Xishuangbanna Tropical Botanical Garden, Chinese Academy of Sciences (101°25′E, 21°41′N), Mengla, Yunnan. Three complete plants (including roots, stems, leaves, and flowers) for genome and transcriptome sequencing.

Vector construction and plant transformation

To perform rice regeneration and transformation assays, the nucleotide acid sequences of LtIAD were synthesized into the pCambia1301s vector by Universe Gene Technology (Tianjin) Co., Ltd. The binary Cambia1301-LtIAD vector was transformed into the Agrobacterium tumefaciens strain EHA105 and then used to genetically transform Nipponbare (*Oryza sativa* L. spp. japonica) callus via the Agrobacterium-mediated transformation method, following the protocol described by Hiei et al (Hiei et al., 1994). Transformed rice plants were grown in a greenhouse in Harbin, China, under controlled conditions of a 14-hour photoperiod at 28 ± 2 °C.

Library construction and sequencing

Genomic DNA was extracted from young leaves using the Novel Plant Genomic DNA Rapid Extraction Kit (Genenode Biotech, Beijing, China) according to the manufacturer's instructions. The extracted genomic DNA was fragmented randomly with an Ultrasonic Crusher, and DNA fragments of specific lengths were purified by electrophoresis. Subsequent steps, including adapter ligation and DNA cluster preparation, were performed prior to sequencing on Illumina HiSeq 2500 platform with PE-100 protocol. Illumina sequencing generated raw genomic data for *L. tsaoko* and *W. villosum* were obtained.

For long-read sequencing, high-quality genomic DNA from *L. tsaoko* was used to construct PacBio libraries with a mean insert size of 52,640 bp, prepared using the SMRT Template Prep Kit (Pacific Biosciences, USA). Sequencing was conducted on the PacBio Sequel platform, and reads with a quality score below 0.8 were excluded. Similarly, genomic DNA libraries for *W. villosum* were sequenced on two cells of the PacBio Sequel platform. Raw data were processed to remove adapters and low-quality reads using SMRT Portal analysis.

Hi-C libraries of *L. tsaoko* and *W. villosum* were prepared from young leaves was also prepared following the standard procedures. The cross-linked and lysed cells were digested with the Dnp II restriction enzyme, and the resulting libraries were sequenced with a paired-end strategy. The raw Hi-C reads were processed using Trimmomatic (version 0.39) (Bolger et al., 2014) to

remove low-quality reads and adaptors. The parameters used were LEADING:20 TRAILING:20 SLIDINGWINDOW:4:20 MINLEN:50, while other parameters were set to default.

RNA Extraction, library construction and Sequencing

Total RNA was extracted using the Hipure HP Plant RNA Mini Kit (Magen, Guangzhou, China), following the manufacturer's instructions. The quality and purity of the RNA samples were assessed using an RNA 6000 Nano LabChip Kit (Agilent Technologies, Santa Clara, CA, USA) on an Agilent Bioanalyzer, ensuring an RNA integrity number (RIN) greater than 7.0. Polyadenylated mRNA was isolated from the total RNA using the NEBNext® Poly(A) mRNA Magnetic Isolation Module (New England Biolabs, Ipswich, MA, USA). The isolated mRNA was fragmented into short fragments using fragmentation buffer, which were then used as templates for the synthesis of first-strand cDNA by reverse transcription. Second-strand cDNA synthesis was performed using the supplied buffer and the Second Strand Synthesis Enzyme Mix. The resulting double-stranded cDNA fragments underwent end-repair and adapter ligation. The uracil bases in the adapters were excised using the USER enzyme, and adapter-ligated fragments were purified via gel electrophoresis. Finally, the purified fragments were amplified by PCR to create the final cDNA library, which was prepared using the NEBNext® Ultra™ RNA Library Prep Kit for Illumina® (New England Biolabs, Ipswich, MA, USA). The resulting libraries were sequenced using Illumina sequencing technology.

Genome assembly and Pseudomolecule Clustering

The PacBio sequencing data were utilized for the *de novo* assembly of *L. tsaoko* genome by NextDenovo2.0 (https://github.com/Nextomics/NextDenovo) with default parameters ([General]: job_type = local job_prefix = nextDenovo task = all rewrite = yes deltmp = yes rerun = 3 parallel_jobs = 30 input_type = raw read_type = clr input_fofn = input.fofn workdir = 01_rundir usetempdir = /tmp/ [correct_option]: read_cutoff = 1k genome_size = 2 g sort_options = -m 20g -t 15 minimap2_options_raw = -t 8 pa_correction = 3 correction_options

= -p 15[assemble_option]: minimap2_options_cns = -t 8 nextgraph_options = -a 1). The Hifiasm (v0.16.0-r369) was applied for *W. villosum* genome assembly by integrating Hi-C data using the parameters '--h1' and '--h2' to specify the Hi-C read files, following the method described by Cheng et al(Cheng et al., 2021). This approach leverages the high-fidelity PacBio reads along with Hi-C data to produce a haplotype-resolved genome assembly. For the two genomes, the next-generation sequencing data were aligned to the draft genome using minimap2 with default parameters, and Purge_dups (Guan et al., 2020) was used to remove the redundant sequence with default parameter. Finally, Illumina sequencing data were performed to polish the assembled sequences with NextPolish (https://github.com/Nextomics/NextPolish/releases/tag/v1.1.0). Benchmarking Universal Single-Copy Orthologs (BUSCO) (Simao et al., 2015) analysis was used to assess the completeness of the genome with the embryophyte_odb10 lineage (Simao *et al.*, 2015).

To improve the quality of genome, the draft assembly contigs were anchored into a Pseudo-chromosome using a Hi-C proximity-based assembly approach. Illumina Hi-C reads were first aligned to the contigs using BWA-MEM (version 0.7.17-r1188) (Li and Durbin, 2009). After alignment, the Juicer pipeline (version 1.5.6) (Durand et al., 2016a)was employed to process the aligned Hi-C data with the parameter -s DpnII, while other parameters were set to default. Juicer helps in extracting valid Hi-C interactions by filtering out noise and artifacts, ensuring that only high-confidence interactions are used for the subsequent assembly steps. The specific commands used were: juicer.sh -g draft_genome -s DpnII -z ./ref/draft.genome.fa -y ./ref/draft.genome_DpnII.txt -p ./ref/draft.genome.chrom.sizes -t 8. The contigs were then ordered and oriented using the 3D-DNA pipeline (version 201008)(Dudchenko et al., 2017) with the parameters -r 3 --sort-output, while other parameters were set to default. This parameter helps in resolving repetitive regions by ensuring sufficient coverage for accurate assembly. The command used was: run-asm-pipeline.sh ./ref/draft.genome.fa ./aligned/merged_nodups.txt -r 0 --sort-output. The resulting Hi-C contact matrix was visualized using Juicebox (version 1.11.08)(Durand et al., 2016b). Based on the visualized Hi-C contact matrix and neighboring interactions, misassemblies and

misjoins were manually corrected. With the finalize section from 3D-DNA, the manually validated assembly was used to build pseudomolecules ordered by size.

Genome annotation and gene expression analysis

The genome annotation of *L. tsaoko* and *W. villosum* was performed via a combination of homology-based, transcriptome-based and de novo prediction. GlimmerHMM (Majoros et al., 2004), SNAP (Korf, 2004), GenScan (Burge and Karlin, 1997) and AUGUSTUS (v3.2.2) (Stanke et al., 2006) were used for ab initio prediction of protein-coding genes with default parameters. The protein sequence from *M. acuminata*, *M. balbisiana*, *Z. officinale* and *O. sativa* were downloaded from NCBI (National Center for Biotechnology Information) database for homology-based prediction. These protein sequences were aligned to *L. tsaoko* and *W. villosum* genome using TBLASTN (v2.2.29+)(Camacho et al., 2009) with a cutoff E-value of 1e-5. The genomic regions of each TBLASTN hit were further evaluated using GeneWise (v2.4.1) (Birney et al., 2004) to predict gene models.

For transcriptome-based gene prediction, RNA-seq raw reads were preprocessed with Trimmomatic (Bolger *et al.*, 2014) to remove adapter sequences and low-quality reads. Clean reads were aligned to the *L. tsaoko* and *W. villosum* genomes using TopHat2 (Trapnell et al., 2009) and assembled the transcripts with Cufflinks (Trapnell et al., 2010) using default parameters, separately. The assembled transcripts were aligned to the genome and analyzed with Program to Assemble Spliced Alignments (PASA) (v2.4.1) (Haas et al., 2008) to generate transcriptome-based gene models. The transcriptome-based gene models of PASA were used to train AUGUSTUS (v3.2.2) with the script 'autoAugTrain.pl'. Subsequently, the transcriptome-trained AUGUSTUS (v3.2.2) was used to predict the structure of protein-coding genes. Finally, the EvidenceModeler (v1.1.1) (Haas *et al.*, 2008) was used to combine the gene models into weighted consensus gene structures with repetitive elements masked. (the following weights: homology-set >transcripts-set>ab initio-set and redundant genes were removed).

Repetitive elements were annotated using RepeatMasker (Tarailo-Graovac and Chen, 2009) (Tarailo-Graovac & Chen, 2009) with the *Viridiplantae* database and RepeatBase (Release16.10; http://www.girinst.org/repbase/index.html) as references. Homologous transposable elements (TEs) were identified via RepeatProteinMask (http://www.repeatmasker.org/). For de novo TE discovery, LTRharvest (Ellinghaus et al., 2008) and LTR_FINDER_parallel (release 09/27/2019) (Xu and Wang, 2007) were used to detect long terminal repeat retrotransposons (LTR-RT) in the genome of *L. tsaoko*. These LTR-RTs were classified and refined using LTR_retriever (version 2.8) (Ou et al., 2018) to generate non-redundant LTR-RT library. Additionally, Repeatmodeler (version 2.0) (http://www.repeatmasker.org/) was applied to construct another de novo repeat library. RepeatMasker was subsequently run again with the merged de novo repeat library to annotate the genome. Tandem repeats were identified with TRF (version 4.07b) (Benson, 1999).

Gene functional annotation was performed by searching against public database using BLASTP. SwissProt (http://www.uniprot.org/), TrEMBL (http://www.uniprot.org/), KEGG (http://www.genome.jp/kegg/), and InterPro (https://www.ebi.ac.uk/interpro/) were used for screening the functional domains of the proteins. Gene Ontology (GO) terms for each gene were extracted from the corresponding InterPro entries.

The HISAT2 was used to build the reference genome index with the default parameters (hisat2-build ref.genome -t 30). The clean reads were then mapped to the reference genome using HISAT2 (Kim et al., 2019) with default parameters. The output was a SAM file, which was converted to a sorted BAM file for subsequent analysis. Stringtie (Pertea et al., 2015) was used to assemble transcripts from the BAM files of each sample. All samples' transcript files were merged into a non-redundant transcript set using StringTie's --merge option. StringTie was used again to estimate transcript abundances. and finally Ballgown (Frazee et al., 2015) was used in the R environment for differential expression analysis. To identify significant differentially expressed genes, we applied the following criteria: genes with a False Discovery Rate (FDR) less than 0.05 and an absolute value of $\log_2$ fold change ($|\log_2 FC|$) greater than 1.

Evolutionary analysis

The genome of *Amborella trichopoda, Nymphaea colorata, Areca catechu, Ananas comosus, Pharus latifolius, O. sativa, Panicum hallii, Musa acuminata, Musa balbisiana* and *Zingiber officinale* were downloaded from JGI/NCBI, and *W. villosum* and *L. tsaoko*, which was assembled in this study, for evolutionary analysis. The orthogroups were produced by OrthoFinder (Emms and Kelly, 2019) with default settings based on the longest mRNA sequences of the species mentioned above. The single-copy genes in 12 vascular plants were concatenated, and the sequences were aligned and trimmed with MAFFT (v6.240) (Katoh and Standley, 2013) and trimAl (v1.2) (Capella-Gutierrez et al., 2009). The divergence times of the species were estimated based on the timetree website using MCMCTREE in the PAML package (Yang, 2007). Based on the calculated phylogeny and divergence time, CAFÉ (v5.0)(De Bie et al., 2006) was used to identify gene families that had undergone expansion or contraction in the above species. KEGG enrichment analysis and GO enrichment analysis were performed using Ontologizer (http://ontologizer.de/) to screen out specific gene families with significant shrinkage and expansion ($p < 0.05$). The p-values were corrected by multiple sampling after calculating the p-values using Thermo Fisher's exact test.

Collinearity and Polyploidy Events of *L. tsaoko* and *W. villosum* genome

Syntenic blocks within *L. tsaoko* and *W. villosum*, as well as between *L. tsaoko*, *W. villosum* and *Z. officinale* were identified based on paralogous or homologous gene pairs using Mcscan (Python version) (https://github.com/tanghaibao/jcvi/wiki/MCscan-(Python-version)). LAST (Kielbasa et al., 2011) was employed to identify the homologs within the *L. tsaoko* genome. Tandem duplications and weak hits were filtered from the resulting gene pairs, and a single-linkage clustering algorithm was applied to the LAST output to cluster anchors into syntenic blocks. To infer whole-genome duplication (WGD) events, synonymous substitution (K_S) values were estimated using the Nei-Gojobori method implemented in the YN00 program within the PAML package (Yang, 2007). Given the varying evolutionary rates across species, K_S values were corrected for more precise identification of WGD events and divergence times

using *ksrates* (Sensalari et al., 2022). First, ksrates was used to estimate the K_S values of one-to-one orthologs and paralogs. Then, rate-adjustment was performed based on the phylogenetic tree with branch lengths equal to the K_S distances estimated from the ortholog K_S distributions.

Resequencing Analysis

A total of 200 *L. tsaoko* plants comprising 98 PG-morph and 99 PA-morph individuals, were selected from the experimental population. Young leaves were collected from each plant, immediately snap-frozen in liquid nitrogen, and used for DNA extraction with the DNAsecure Plant Kit (Tiangen, Beijing, China). Genomic DNA (2 μg per sample) was used to construct sequencing libraries with the NEBNext® Ultra™ II DNA Library Prep Kit for Illumina (NEB, USA). Paired-end libraries with an average insert size of approximately 400 bp were sequenced on the Illumina HiSeq 4000 platform at Novogene (Beijing, China).

After quality control and filtering of the raw sequencing data, the clean reads were aligned to the reference genome with BWA. Apply Samtools (Danecek et al., 2021), Reseqtools (He et al., 2013) and Picard-tools (https://broadinstitute.github.io/picard/) to compare the results for statistics and preprocessing (sorting, deduplication, adding ID, etc.). SNP and Indel information were detected with GATK3.8 (McKenna et al., 2010). High-confidence SNPs and Indels were obtained by filtering polymorphic sites between the tested genotypes and the reference genome. For genotype data processing, SNPs were filtered based on a minor allele frequency (MAF) threshold of 5% and a maximum missing rate of 10%. Genotype imputation was carried out using Beagle software, resulting in the final genotype dataset. LD-based filtering of variant sites was performed with Plink software (Purcell et al., 2007) using parameters of a window size of 50, step size of 50, and $r^2 \geq 0.2$. The total number of valid SNP sites was calculated, and association analyses were conducted using GEMMA (Zhou and Stephens, 2012) with mixed linear models (MLM).The significance threshold (Bonferroni threshold) corresponding to the two *P* values, that is, the value of $-\log_{10}P$, was used as the subsequent selection criteria to screen more and more accurate candidate genes. The software performs association analysis based on mixed linear models (GLM) with GEMMA

RNA extraction and real-time quantitative polymerase chain reaction (RT-qPCR) and RT-PCR

To detect the transcript level of *LtIAD* in transgenic rice, the rice buds were collected to extracted total RNA. The total RNA of all rice samples was extracted using a plant RNA isolation reagent (Quick RNA Isolation Kit was purchased from Huayueyang Biotechnology Co., Ltd., China.), and RNA was reverse-transcribed into cDNA with TransScript One-Step gDNA Removal and cDNA Synthesis SuperMix (Transgen Biotech, Cat#AU311-02) according to the manufacturer's instructions. Expression levels were normalized to the OsUBQ control gene and calculated by the $2^{-\blacktriangle CT}$ method. Four independent technical replicates were performed. All primers are listed in Supplementary Table S22.

Staining of anthers and measure of dehiscent area

Rice flower buds were collected according for three flowering stages, and fixed in Carnoy's fixative (6 alcohol:3 chloroform:1 acetic acid) for a minimum of 2 hours. The stain solution we utilized, following Ross's method, consisted of 9.5% alcohol, 0.01% malachite green, 25% glycerol, 0.05% acid fuchsin, 0.005% orange G, 4% glacial acetic acid. After fixation, the bud should be dissected to release the anthers and placed on a microscope slide. Apply 2-4 drops of the stain solution before the sample completely dries. Each anther was photographed using a light microscope. The ImageJ program was applied to calculate the area of dehiscent area.

$$\text{Anther dehiscent ratio}=\frac{\text{Anther area-Pollen area}}{\text{Anther area}} * 100\%$$

TWAS, eQTL and Co-expression network analysis

To identify genes involved in the process of dichogamy in *L. tsaoko*, we obtained 99 PA morph and 98 PG morph whole-flower transcriptomes respectively. Differential gene expression analysis was performed, followed by the removal of low-expressed genes. Transcriptome-wide association study (TWAS) analysis was conducted on the differentially expressed genes, and

the resulting expression matrices were used for subsequent analyses. Correlation analyses were performed using linear and mixed linear models implemented in EMMAX (Kang et al., 2010), with an FDR-corrected p-value threshold of ≤ 0.01 to determine significance. Given the potential for false positives in TWAS results, we conducted additional analyses to identify expression quantitative trait loci (eQTLs) associated with significant SNP loci identified through GWAS. eQTL analysis was performed using MatrixEQTL (version 2.3) (Shabalin, 2012) with SNP matrices. Significance thresholds of $p \leq 0.01$ were applied for both cis- and trans-eQTLs. TWAS results were cross-referenced with these eQTLs to obtain a set of reliable candidate genes for further analysis.

Subsequently to identify the genes that play major roles in the process of dichogamy, the R package WGCNA (version 1.72-1)(Langfelder and Horvath, 2008) was used to construct the WGCNA co-expression network based on the expression matrix of all the genes with 176 candidate genes as the core, which was expanded outward by 1 node after taking the intersection with the total expression network, and filtered by Weight ≥ 0.2 to obtain the final WCGNA co-expression network. Finally, Cytoscape (version 3.10.1) (Shannon et al., 2003) was used for visualization. Emapper (version 2.1.12) (Cantalapiedra et al., 2021) was used to functionally annotate the protein sequences of the target genes. The gene from the network were aligned with the protein sequences of *Arabidopsis thaliana* with DIAMOND (v2.0.14)(Buchfink et al., 2021) with default parameters. The target genes were annotated with the description entries of the *A. thaliana* genes to identify homologues and their possible functions.

Benson, G. (1999). Tandem repeats finder: a program to analyze DNA sequences. Nucleic Acids Research **27**:573-580. 10.1093/nar/27.2.573.

Birney, E., Clamp, M., and Durbin, R. (2004). GeneWise and genomewise. Genome Research **14**:988-995. 10.1101/gr.1865504.

Bolger, A.M., Lohse, M., and Usadel, B. (2014). Trimmomatic: a flexible trimmer for Illumina sequence data. Bioinformatics **30**:2114-2120. 10.1093/bioinformatics/btu170.

Buchfink, B., Reuter, K., and Drost, H.-G. (2021). Sensitive protein alignments at tree-of-life scale using DIAMOND. Nature Methods **18**:366-+. 10.1038/s41592-021-01101-x.

Burge, C., and Karlin, S. (1997). Prediction of complete gene structures in human genomic DNA. Journal of Molecular Biology **268**:78-94. 10.1006/jmbi.1997.0951.

Camacho, C., Coulouris, G., Avagyan, V., Ma, N., Papadopoulos, J., Bealer, K., and Madden, T.L. (2009). BLAST plus : architecture and applications. Bmc Bioinformatics **10**10.1186/1471-2105-10-421.

Cantalapiedra, C.P., Hernandez-Plaza, A., Letunic, I., Bork, P., and Huerta-Cepas, J. (2021). eggNOG-mapper v2: Functional Annotation, Orthology Assignments, and Domain Prediction at the Metagenomic Scale. Molecular Biology and Evolution **38**:5825-5829. 10.1093/molbev/msab293.

Capella-Gutierrez, S., Silla-Martinez, J.M., and Gabaldon, T. (2009). trimAl: a tool for automated alignment trimming in large-scale phylogenetic analyses. Bioinformatics **25**:1972-1973. 10.1093/bioinformatics/btp348.

Cheng, H., Concepcion, G.T., Feng, X., Zhang, H., and Li, H. (2021). Haplotype-resolved de novo assembly using phased assembly graphs with hifiasm. Nature Methods **18**:170-+. 10.1038/s41592-020-01056-5.

Danecek, P., Bonfield, J.K., Liddle, J., Marshall, J., Ohan, V., Pollard, M.O., Whitwham, A., Keane, T., McCarthy, S.A., Davies, R.M., et al. (2021). Twelve years of SAMtools and BCFtools. Gigascience **10**10.1093/gigascience/giab008.

De Bie, T., Cristianini, N., Demuth, J.P., and Hahn, M.W. (2006). CAFE: a computational tool for the study of gene family evolution. Bioinformatics **22**:1269-1271. 10.1093/bioinformatics/btl097.

Dudchenko, O., Batra, S.S., Omer, A.D., Nyquist, S.K., Hoeger, M., Durand, N.C., Shamim, M.S., Machol, I., Lander, E.S., Aiden, A.P., et al. (2017). De novo assembly of the Aedes aegypti genome using Hi-C yields chromosome-length scaffolds. Science **356**:92-95. 10.1126/science.aal3327.

Durand, N.C., Shamim, M.S., Machol, I., Rao, S.S.P., Huntley, M.H., Lander, E.S., and Aiden, E.L. (2016a). Juicer Provides a One-Click System for Analyzing Loop-Resolution Hi-C Experiments. Cell Systems **3**:95-98. 10.1016/j.cels.2016.07.002.

Durand, N.C., Robinson, J.T., Shamim, M.S., Machol, I., Mesirov, J.P., Lander, E.S., and Aiden, E.L. (2016b). Juicebox Provides a Visualization System for Hi-C Contact Maps with Unlimited Zoom. Cell Systems **3**:99-101. 10.1016/j.cels.2015.07.012.

Ellinghaus, D., Kurtz, S., and Willhoeft, U. (2008). LTRharvest, an efficient and flexible software for de novo detection of LTR retrotransposons. Bmc Bioinformatics **9**10.1186/1471-2105-9-18.

Emms, D.M., and Kelly, S. (2019). OrthoFinder: phylogenetic orthology inference for comparative genomics. Genome Biology **20**10.1186/s13059-019-1832-y.

Frazee, A.C., Pertea, G., Jaffe, A.E., Langmead, B., Salzberg, S.L., and Leek, J.T. (2015). Ballgown bridges the gap between transcriptome assembly and expression analysis. Nature Biotechnology **33**:243-246. 10.1038/nbt.3172.

Guan, D., McCarthy, S.A., Wood, J., Howe, K., Wang, Y., and Durbin, R. (2020). Identifying and removing haplotypic duplication in primary genome assemblies. Bioinformatics **36**:2896-2898. 10.1093/bioinformatics/btaa025.

Haas, B.J., Salzberg, S.L., Zhu, W., Pertea, M., Allen, J.E., Orvis, J., White, O., Buell, C.R., and Wortman, J.R. (2008). Automated eukaryotic gene structure annotation using EVidenceModeler and the program to assemble spliced alignments. Genome Biology **9**10.1186/gb-2008-9-1-r7.

He, W., Zhao, S., Liu, X., Dong, S., Lv, J., Liu, D., Wang, J., and Meng, Z. (2013). ReSeqTools: an integrated toolkit for large-scale next-generation sequencing based resequencing analysis. Genetics and Molecular Research **12**:6275-6283. 10.4238/2013.December.4.15.

Hiei, Y., Ohta, S., Komari, T., and Kumashiro, T. (1994). Efficient transformation of rice (Oryza sativa L.) mediated by Agrobacterium and sequence analysis of the boundaries of the T-DNA. The Plant Journal **6**:271-282.

Kang, H.M., Sul, J.H., Service, S.K., Zaitlen, N.A., Kong, S.-y., Freimer, N.B., Sabatti, C., and Eskin, E. (2010). Variance component model to account for sample structure in genome-wide association studies. Nature Genetics **42**:348-U110. 10.1038/ng.548.

Katoh, K., and Standley, D.M. (2013). MAFFT Multiple Sequence Alignment Software Version 7: Improvements in Performance and Usability. Molecular Biology and Evolution **30**:772-780. 10.1093/molbev/mst010.

Kielbasa, S.M., Wan, R., Sato, K., Horton, P., and Frith, M.C. (2011). Adaptive seeds tame genomic sequence comparison. Genome Research **21**:487-493. 10.1101/gr.113985.110.

Kim, D., Paggi, J.M., Park, C., Bennett, C., and Salzberg, S.L. (2019). Graph-based genome alignment and genotyping with HISAT2 and HISAT-genotype. Nature Biotechnology **37**:907-+. 10.1038/s41587-019-0201-4.

Korf, I. (2004). Gene finding in novel genomes. Bmc Bioinformatics **5**10.1186/1471-2105-5-59.

Langfelder, P., and Horvath, S. (2008). WGCNA: an R package for weighted correlation network analysis. Bmc Bioinformatics **9**10.1186/1471-2105-9-559.

Li, H., and Durbin, R. (2009). Fast and accurate short read alignment with Burrows-Wheeler transform. Bioinformatics **25**:1754-1760. 10.1093/bioinformatics/btp324.

Majoros, W.H., Pertea, M., and Salzberg, S.L. (2004). TigrScan and GlimmerHMM: two open source ab initio eukaryotic gene-finders. Bioinformatics **20**:2878-2879. 10.1093/bioinformatics/bth315.

McKenna, A., Hanna, M., Banks, E., Sivachenko, A., Cibulskis, K., Kernytsky, A., Garimella, K., Altshuler, D., Gabriel, S., Daly, M., et al. (2010). The Genome Analysis Toolkit: A MapReduce framework for analyzing next-generation DNA sequencing data. Genome Research **20**:1297-1303. 10.1101/gr.107524.110.

Ou, S., Chen, J., and Jiang, N. (2018). Assessing genome assembly quality using the LTR Assembly Index (LAI). Nucleic Acids Research **46**10.1093/nar/gky730.

Pertea, M., Pertea, G.M., Antonescu, C.M., Chang, T.-C., Mendell, J.T., and Salzberg, S.L. (2015). StringTie enables improved reconstruction of a transcriptome from RNA-seq reads. Nature Biotechnology **33**:290-+. 10.1038/nbt.3122.

Purcell, S., Neale, B., Todd-Brown, K., Thomas, L., Ferreira, M.A.R., Bender, D., Maller, J., Sklar, P., de Bakker, P.I.W., Daly, M.J., et al. (2007). PLINK: A tool set for whole-genome association and population-based linkage analyses. American Journal of Human Genetics **81**:559-575. 10.1086/519795.

Sensalari, C., Maere, S., and Lohaus, R. (2022). ksrates: positioning whole-genome duplications relative to speciation events in K-S distributions. Bioinformatics **38**:530-532. 10.1093/bioinformatics/btab602.

Shabalin, A.A. (2012). Matrix eQTL: ultra fast eQTL analysis via large matrix operations. Bioinformatics **28**:1353-1358. 10.1093/bioinformatics/bts163.

Shannon, P., Markiel, A., Ozier, O., Baliga, N.S., Wang, J.T., Ramage, D., Amin, N., Schwikowski, B., and Ideker, T. (2003). Cytoscape: A software environment for integrated models of biomolecular interaction networks. Genome Research **13**:2498-2504. 10.1101/gr.1239303.

Simao, F.A., Waterhouse, R.M., Ioannidis, P., Kriventseva, E.V., and Zdobnov, E.M. (2015). BUSCO: assessing genome assembly and annotation completeness with single-copy orthologs. Bioinformatics **31**:3210-3212. 10.1093/bioinformatics/btv351.

Stanke, M., Keller, O., Gunduz, I., Hayes, A., Waack, S., and Morgenstern, B. (2006). AUGUSTUS: ab initio prediction of alternative transcripts. Nucleic Acids Research **34**:W435-W439. 10.1093/nar/gkl200.

Tarailo-Graovac, M., and Chen, N. (2009). Using RepeatMasker to identify repetitive elements in genomic sequences. Current protocols in bioinformatics **Chapter 4**:4.10.11-14.10.14. 10.1002/0471250953.bi0410s25.

Trapnell, C., Pachter, L., and Salzberg, S.L. (2009). TopHat: discovering splice junctions with RNA-Seq. Bioinformatics **25**:1105-1111. 10.1093/bioinformatics/btp120.

Trapnell, C., Williams, B.A., Pertea, G., Mortazavi, A., Kwan, G., van Baren, M.J., Salzberg, S.L., Wold, B.J., and Pachter, L. (2010). Transcript assembly and quantification by RNA-Seq reveals unannotated transcripts and isoform switching during cell differentiation. Nature Biotechnology **28**:511-U174. 10.1038/nbt.1621.

Xu, Z., and Wang, H. (2007). LTR_FINDER: an efficient tool for the prediction of full-length LTR retrotransposons. Nucleic Acids Research **35**:W265-W268. 10.1093/nar/gkm286.

Yang, Z. (2007). PAML 4: phylogenetic analysis by maximum likelihood. Molecular biology and evolution **24**:1586-1591.

Zhou, X., and Stephens, M. (2012). Genome-wide efficient mixed-model analysis for association studies. Nature Genetics **44**:821-U136. 10.1038/ng.2310.

Supplemental Figures

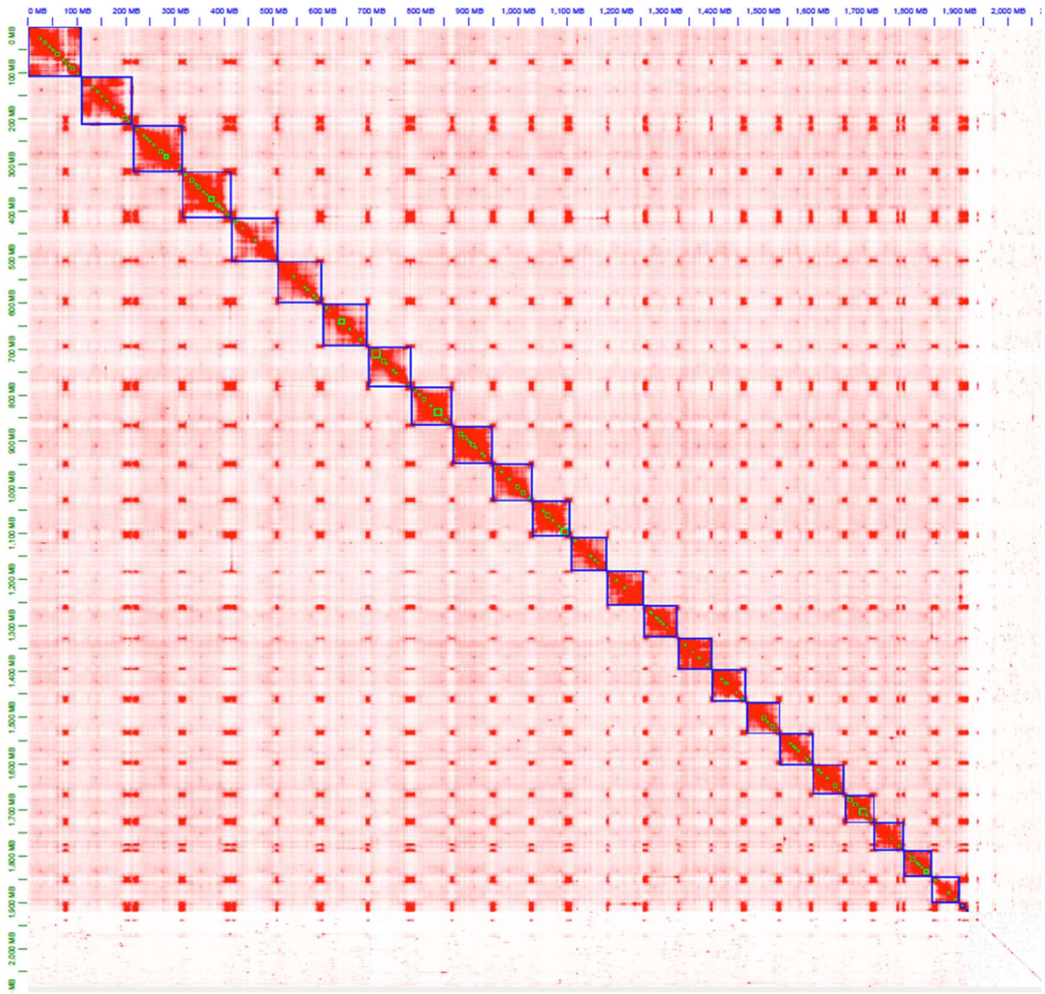

Supplemental Figure 1. Heatmap of the Hi-C interaction density between 24 pseudochromosomes of *L. tsaoko*. The color from light to dark indicates the increase in the intensity of interaction.

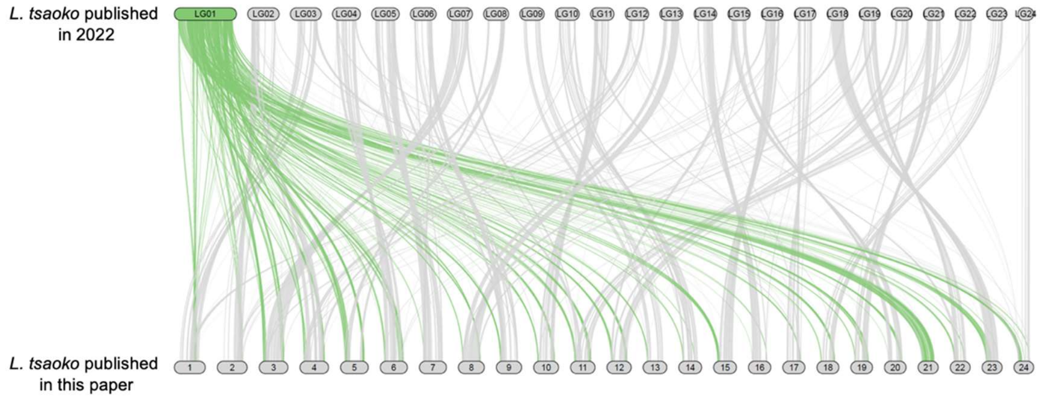

Supplemental Figure 2. Syntenic analysis of the published *L. tsaoko* genome and the genome assembled in this article.

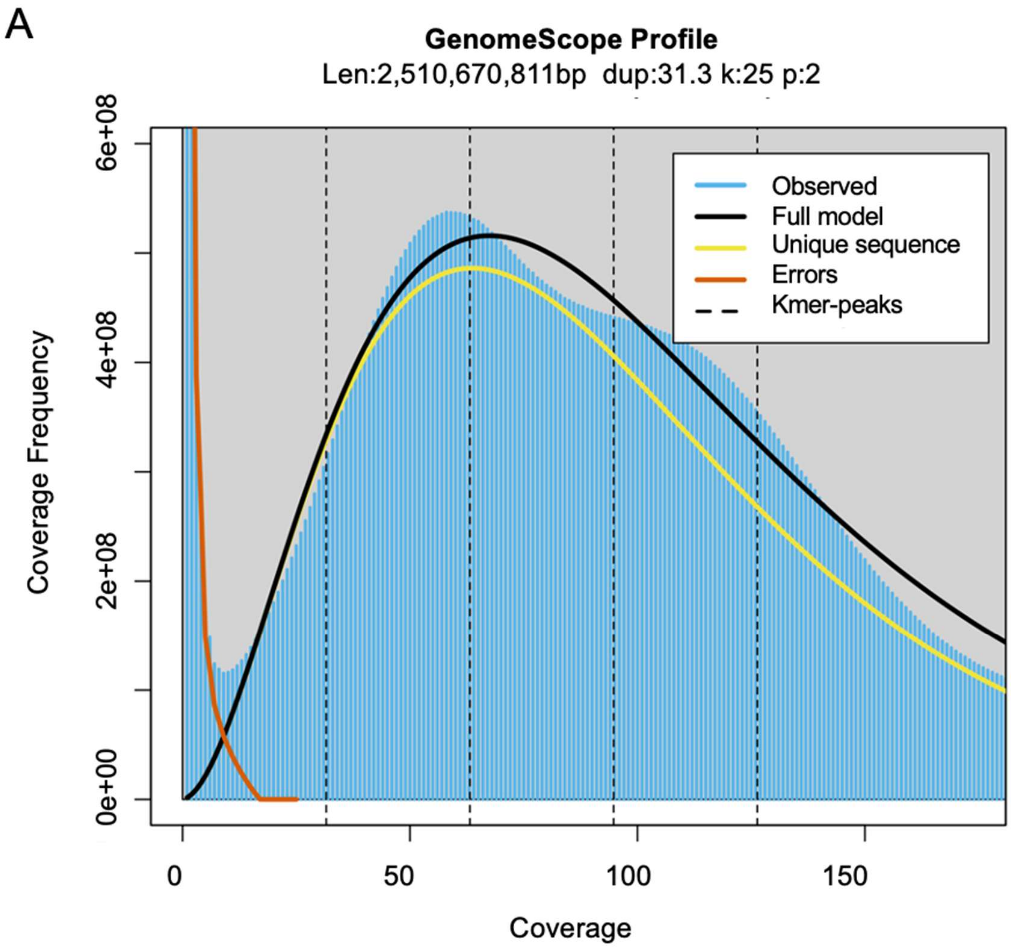

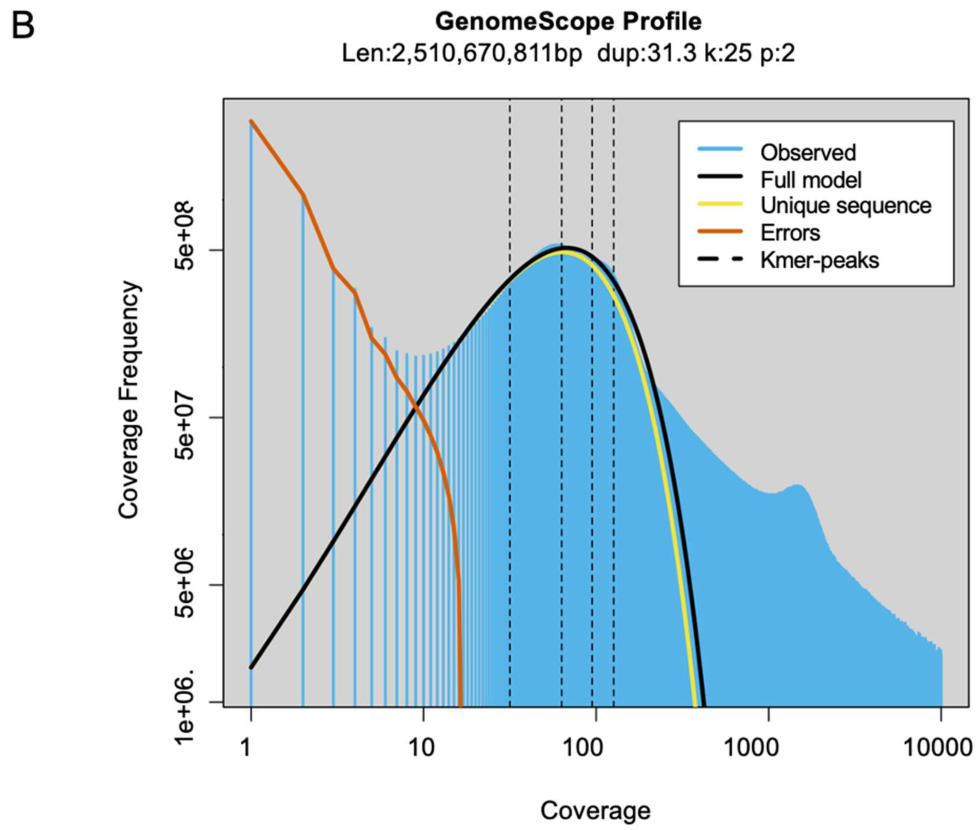

Supplemental Figure 3. The survey analysis to estimate the *W. villosum* genome size. (A-B) Genome size estimation of *W. villosum* genome using *k*-mer distribution (k=25).

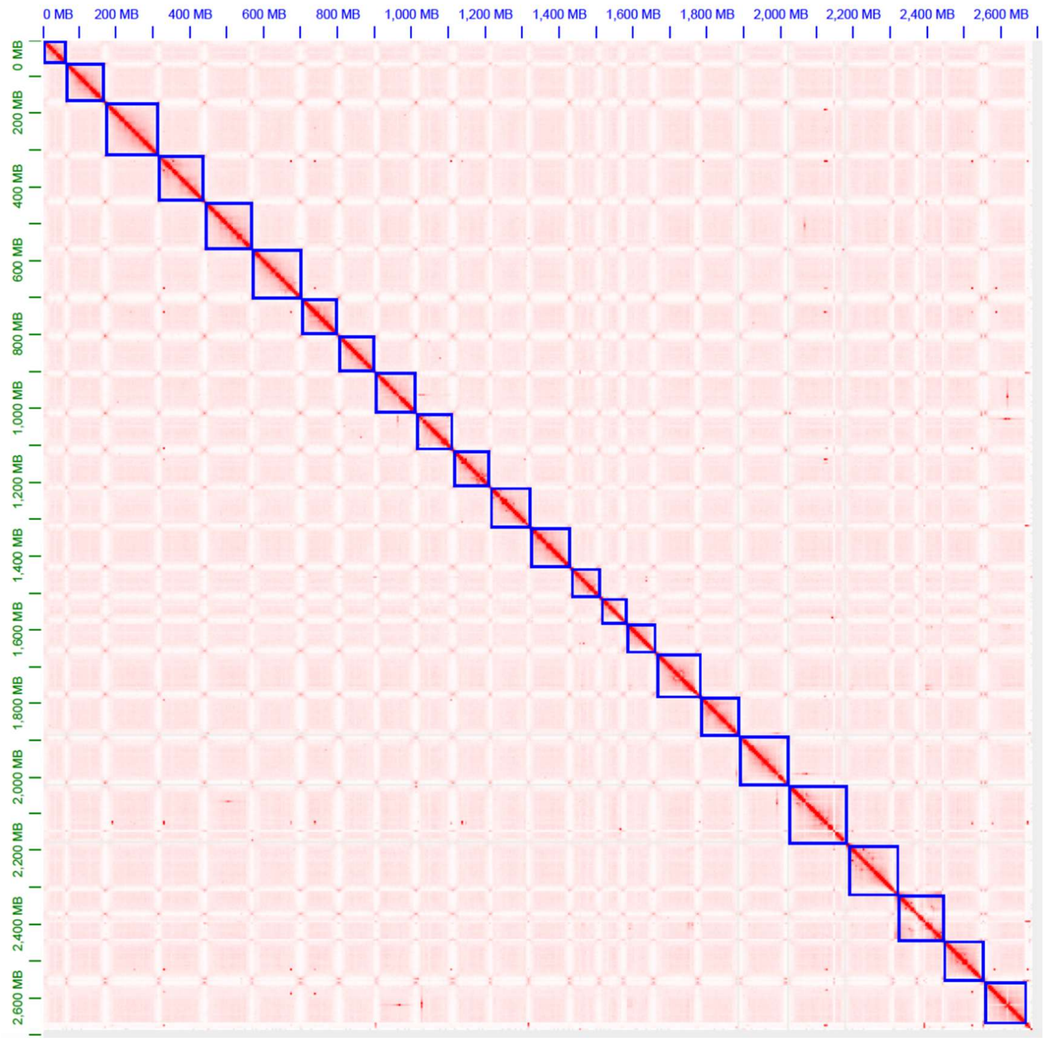

Supplemental Figure 4. Heatmap of the Hi-C interaction density between 24 pseudochromosomes of *W. villosum*. The color from light to dark indicates the increase in the intensity of interaction.

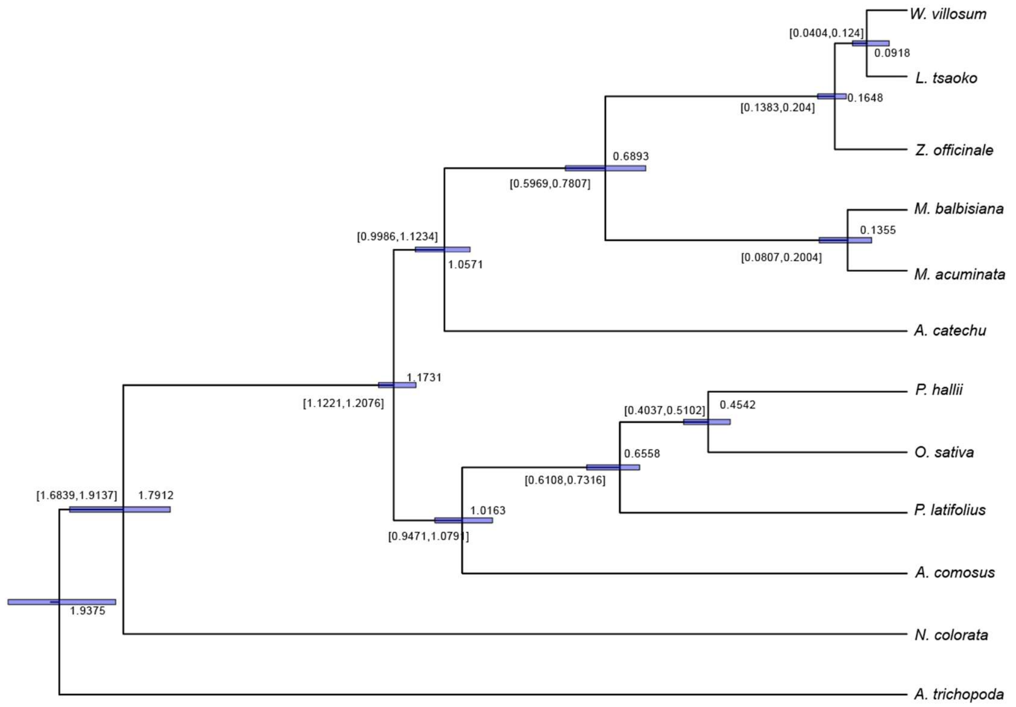

Supplemental Figure 5. Estimation of divergence times for candidate species using MCMCtree. Blue bars represent the 95% Confidence Interval (CI) for divergence times.

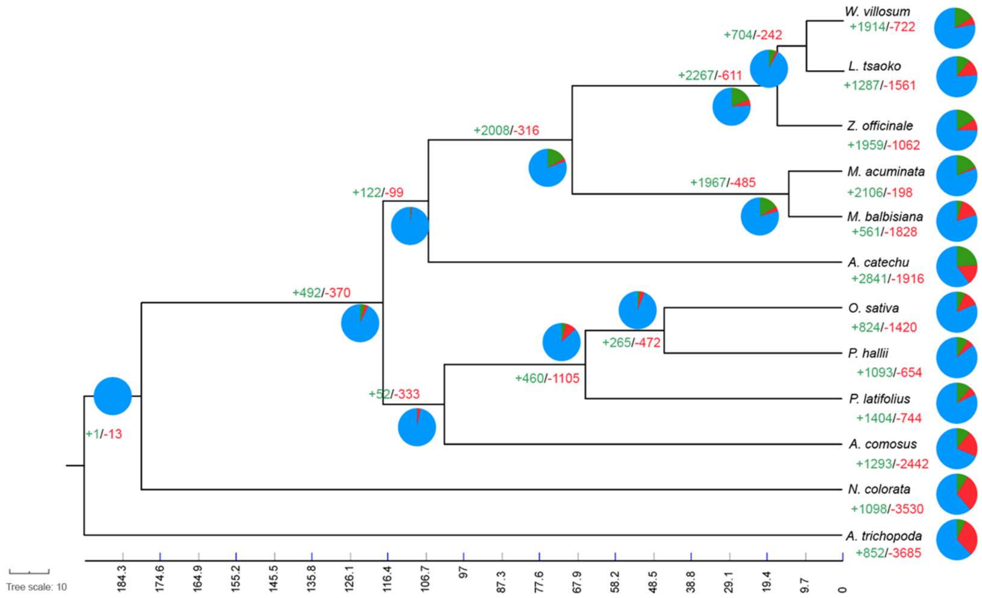

Supplemental Figure 6. Expansion and contraction of gene families in 12 angiosperm species using CAFE5. The occurrence of gene family expansion and contraction is indicated by the

green and red numbers with plus and minus signs, respectively.

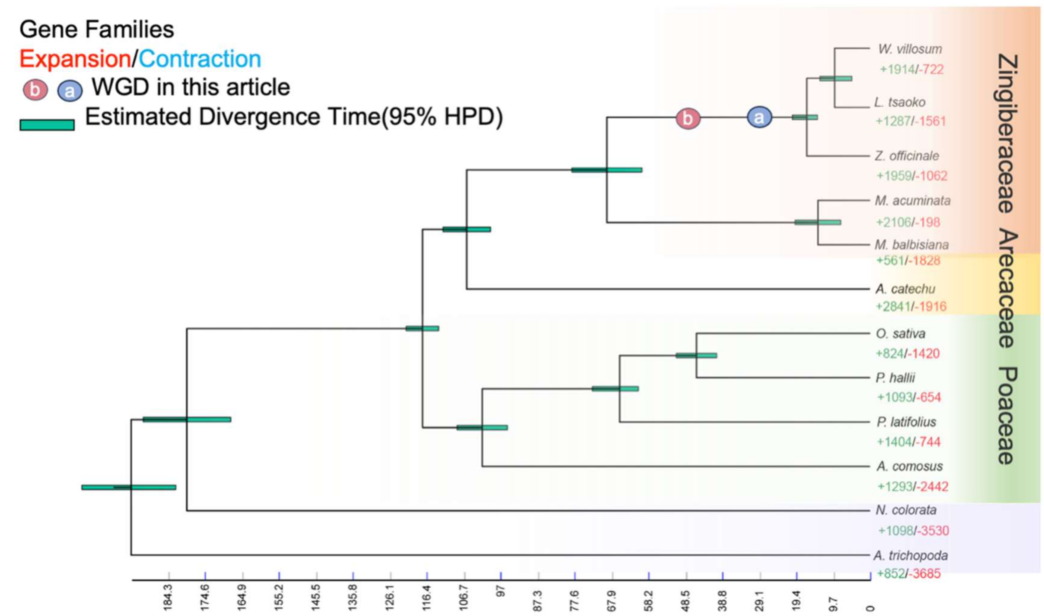

Supplemental Figure 7. Phylogenetic relationship and WGD events of *L. tsaoko*. The occurrence of gene family expansion and contraction is indicated by the green and red numbers with plus and minus signs, respectively. Blue bars at nodes represent 95% credibility intervals of the estimated dates.

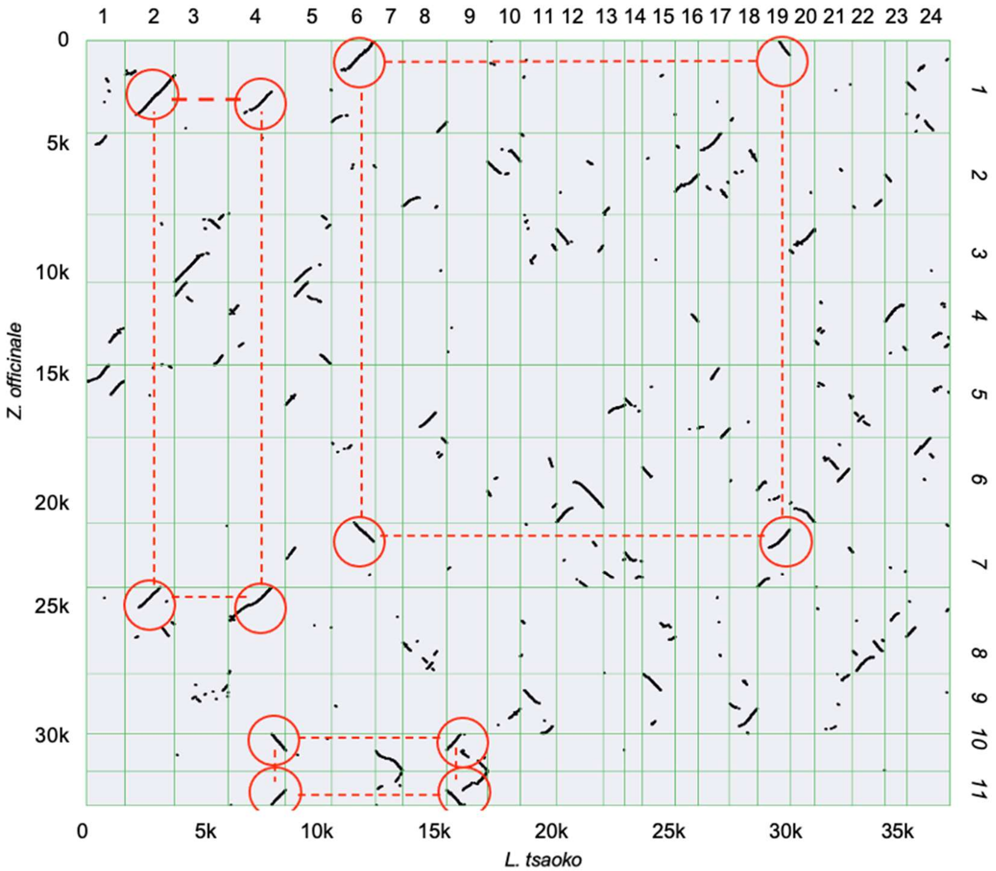

Supplemental Figure 8. Synteny blocks between *L. tsaoko* and *Z. officinale.* The red circles show a 2:2 syntenic relationship between *L. tsaoko* and *Z. officinale*

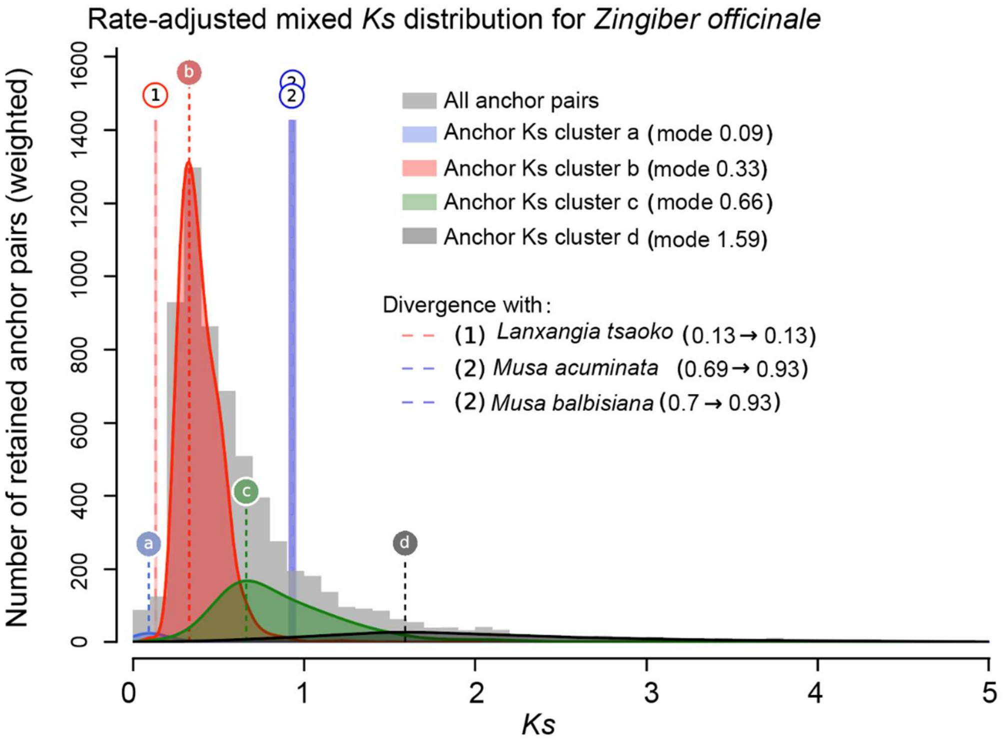

Supplemental Figure 9. K_S distributions of whole paralogs (grey bars) and anchor-pair paralogs (black bars) for *Z. officinale*. Two obvious peaks in the $\log_{10}$ (K_S) distributions indicated that there are at least two WGD events occurred during the evolution of *Z. officinale.*

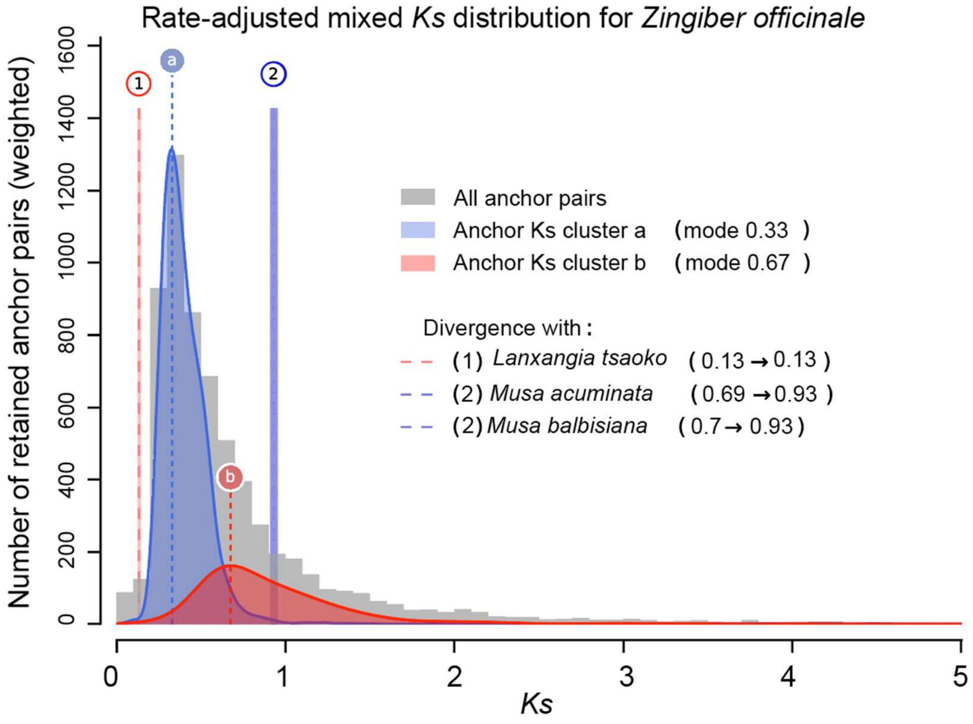

Supplemental Figure 10. Distribution of synonymous substitution rates (K_S) for the anchored paralogs of *Z. officinale* and the anchored orthologs between *Z. officinale* and *L. tsaoko* and *M. acuminata, M. balbisiana*. The colored dashed lines with letters (a, b) represent the WGD events, and the colored dashed lines with numbers (1, 2) represent the divergences of *Z. officinale* with other species.

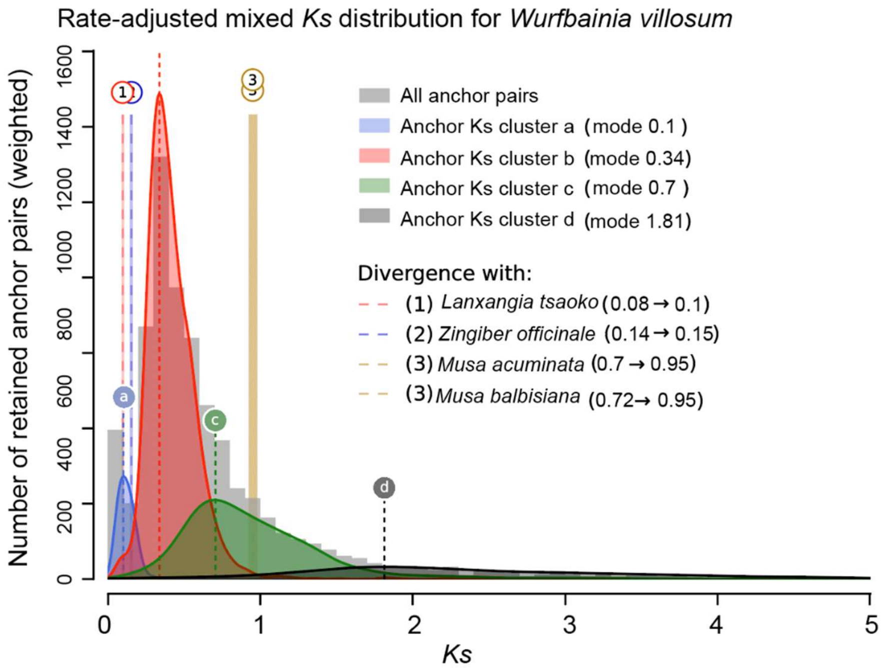

Supplemental Figure 11. *K*S distributions of whole paralogs (grey bars) and anchor-pair paralogs (black bars) for *W. villosum*. Two obvious peaks in the $\log_{10}$ (*K*S) distributions indicated that there are at least two WGD events occurred during the evolution of *W. villosum.*

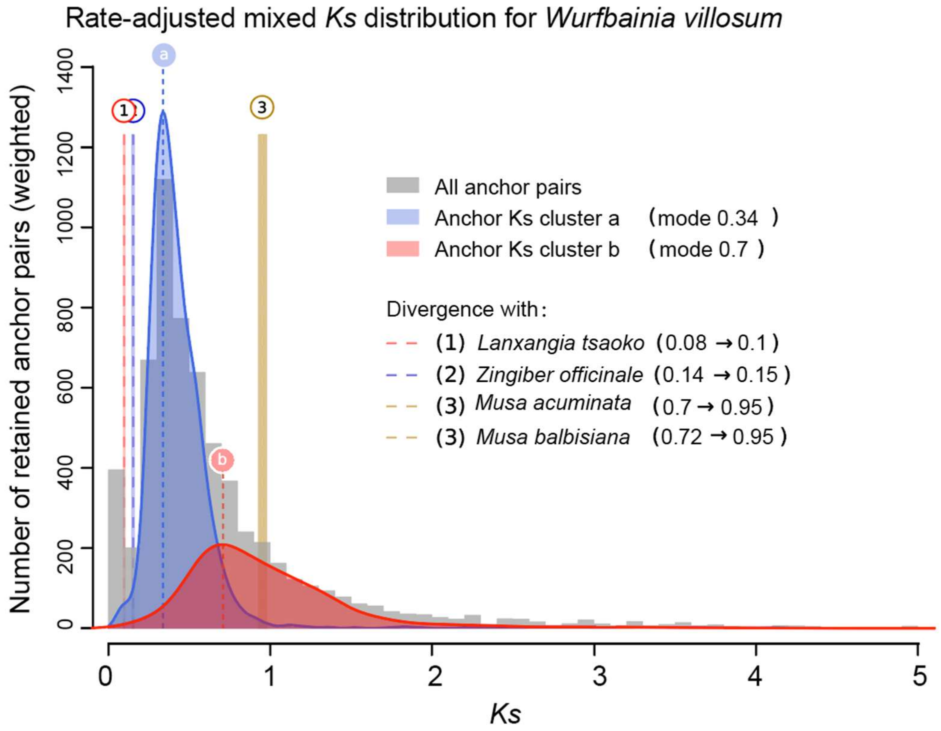

Supplemental Figure 12. Substitution-rate-adjusted mixed paralog-ortholog synonymous substitutions per synonymous site (K_S) plot for *W. villosum* and the anchored orthologs between *W. villosum* and *Z. officinale*, *L. tsaoko* and *M. acuminata, M. balbisiana*. The colored dashed lines with letters (a, b) represent the WGD events, and the colored dashed lines with numbers (1, 2) represent the divergences of *W. villosum* with other species.

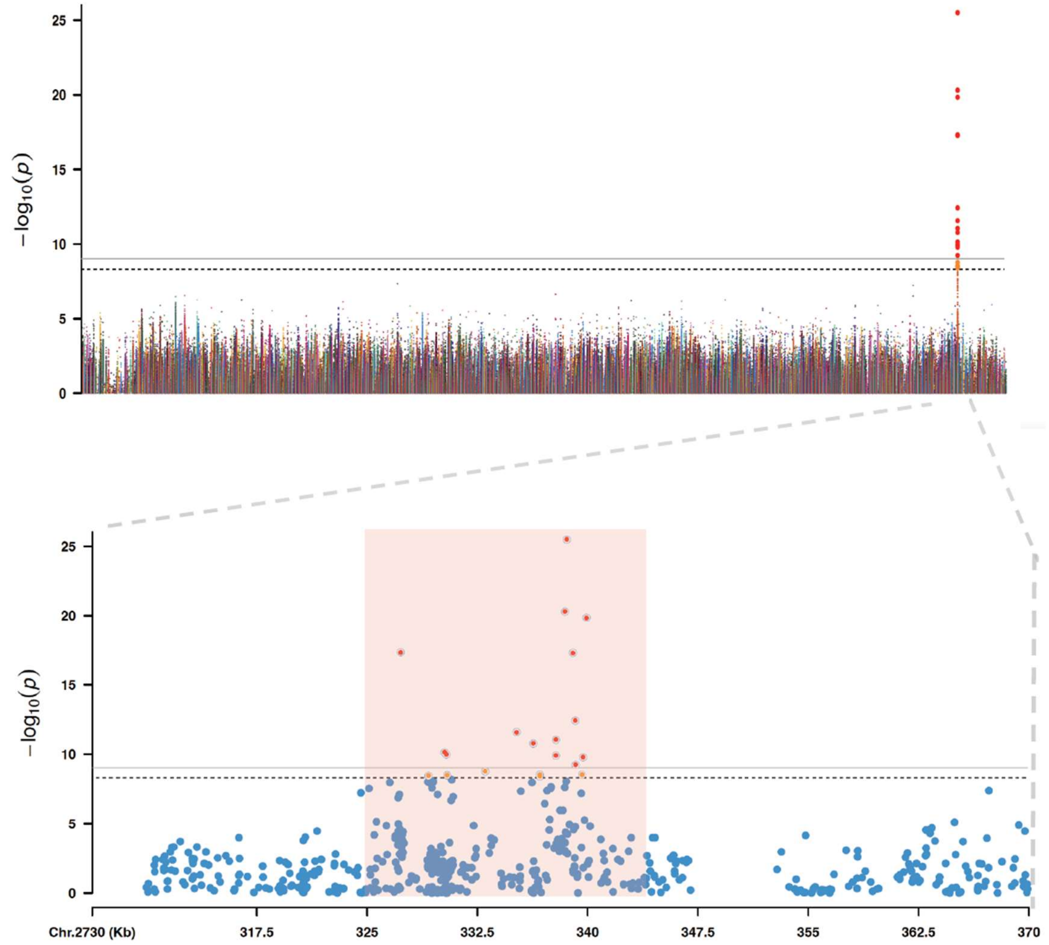

Supplemental Figure 13. Manhattan plot of genome-wide SNP markers associated with dichogamy. The strongest associated SNP on contig-002730F was located. Dashed horizontal black straight and dotted lines indicated significant p-value threshold of 9.91(calculated as 0.01/n)**.** In the red squares are regions with significant correlations.

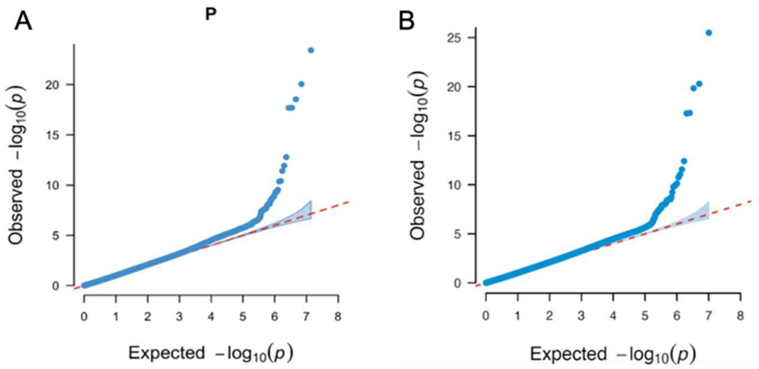

Supplemental Figure 14. QQ plot of genome-wide SNP markers associated with dichogamy.

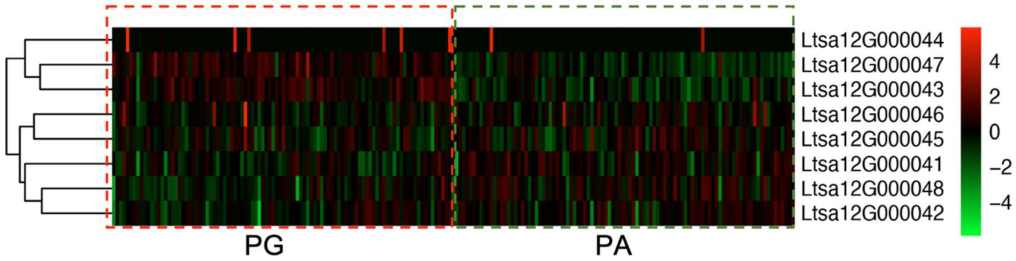

Supplemental Figure 15. Heatmap of gene expression in the *L. tsaoko* - DAR region of *L. tsaoko* genome.

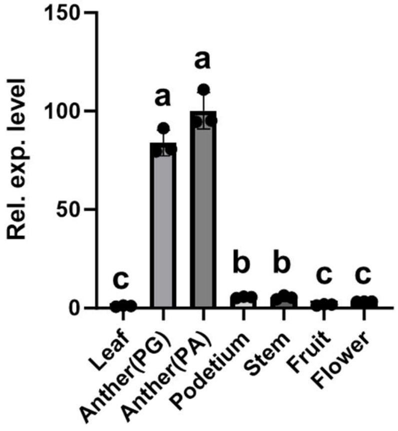

Supplemental Figure 16. Expression profiling of *LtIAD* in different tissues of *L. tsaoko*.

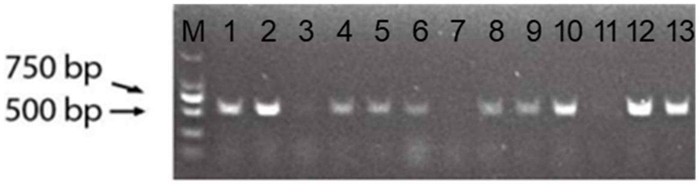

Supplemental Figure 17. Identification of *LtIAD* overexpression lines by hygromycin （hygromycin amplified fragment size was 557 bp, M: D2000 Marker）.

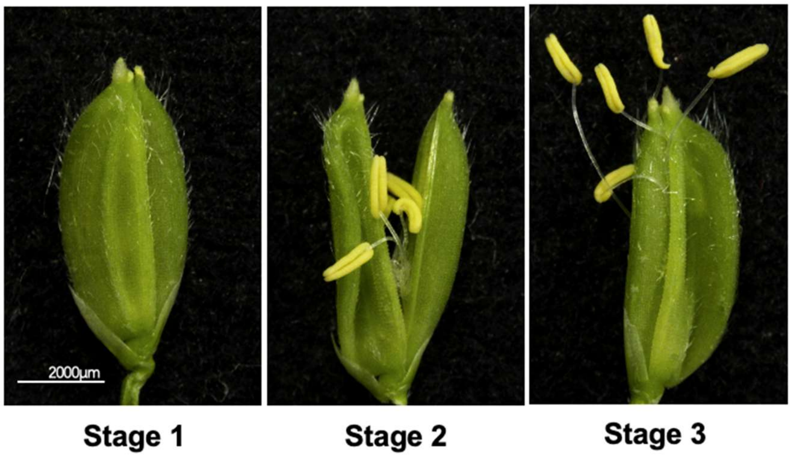

Supplemental Figure 18. The flowering stage are divided into stage 1, stage 2, and stage 3 according to the open condition of palea and lemmal.

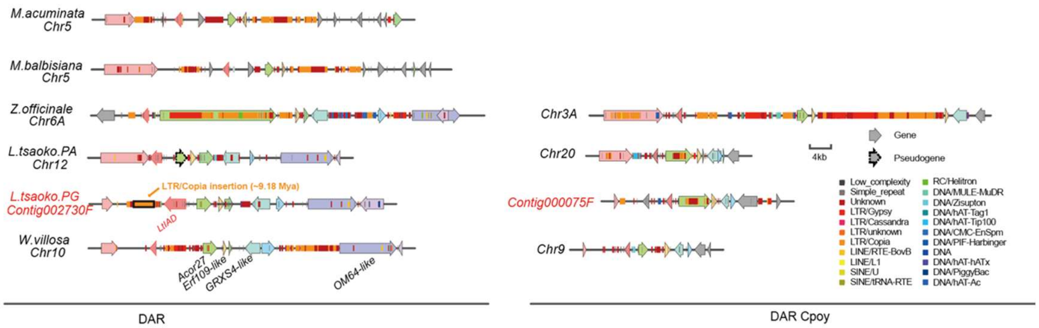

Supplemental Figure 19. The evolution of DARs in the six genomes. Different coloured rectangular squares represent different types of turntables, rectangular squares with arrows represent genes， genes of the same colour represent genes with collinearity.

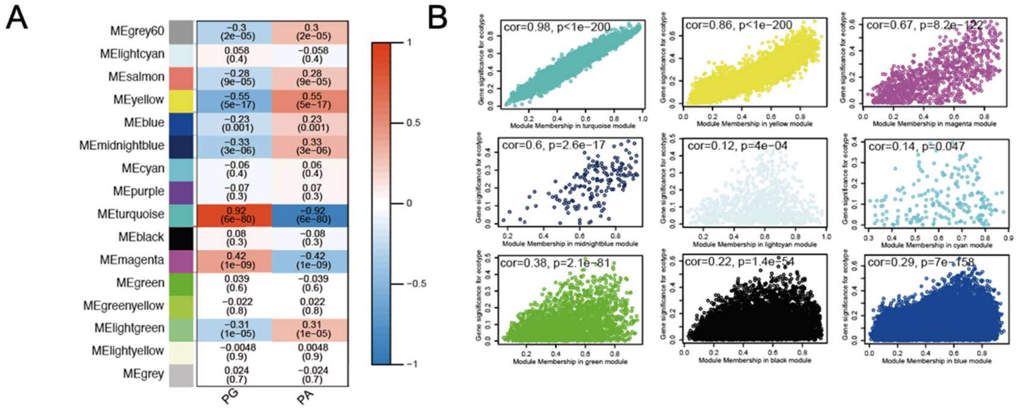

Supplemental Figure 20. A. Module-trait (ecotype) association. Each row corresponds to a module (ME). Each column corresponds to a specific cell phase. Each cell contains a corresponding correlation and P-value of modules with various phases. The color of each cell at the row-column intersection indicates the correlation coefficient between the module and the phase. **B.** Scatter plot of correlation analysis of MEturquoise and ecotype. The higher the correlation between the module MM and the current GS, the more positively the module was correlated with the ecotype.

Supplementary tables. See the accompanying Excel file for details.
